# Cyst(e)ine in nutrition formulation promotes colon cancer growth and chemoresistance by activating mTORC1 and scavenging ROS

**DOI:** 10.1038/s41392-021-00581-9

**Published:** 2021-05-28

**Authors:** Jiao Wu, Sai-Ching Jim Yeung, Sicheng Liu, Aiham Qdaisat, Dewei Jiang, Wenli Liu, Zhuo Cheng, Wenjing Liu, Haixia Wang, Lu Li, Zhongmei Zhou, Rong Liu, Chuanyu Yang, Ceshi Chen, Runxiang Yang

**Affiliations:** 1 Department of the Second Medical Oncology, The Third Affiliated Hospital of Kunming Medical University, Kunming, Yunnan Province China; 2 Department of Emergency Medicine, The University of Texas MD Anderson Cancer Center, Houston, Texas USA; 3 Key Laboratory of Animal Models and Human Disease Mechanisms of Chinese Academy of Sciences and Yunnan Province, Kunming Institute of Zoology, Chinese Academy of Sciences, Kunming, China; 4 Department of Palliative, Rehabilitation and Integrative Medicine, The University of Texas MD Anderson Cancer Center, Houston, Texas USA; 5 Department of Medical Oncology, Sun Yat-sen University Cancer Center, Guangzhou, Guangdong China; 6 KIZ-CUHK Joint Laboratory of Bioresources and Molecular Research in Common Diseases, Kunming Institute of Zoology, Chinese Academy of Sciences, Kunming, China; 7 Institute of Translation Medicine, Shenzhen Second People’s Hospital, The First Affiliated Hospital of Shenzhen University, Shenzhen, China

**Keywords:** Gastrointestinal cancer, Therapeutics

## Abstract

Weight loss and cachexia are common problems in colorectal cancer patients; thus, parenteral and enteral nutrition support play important roles in cancer care. However, the impact of nonessential amino acid components of nutritional intake on cancer progression has not been fully studied. In this study, we discovered that gastrointestinal cancer patients who received cysteine as part of the parenteral nutrition had shorter overall survival (*P* < 0.001) than those who did not. Cystine indeed robustly promotes colon cancer cell growth in vitro and in immunodeficient mice, predominately by inhibiting *SESN2* transcription via the GCN2-ATF4 axis, resulting in mTORC1 activation. mTORC1 inhibitors Rapamycin and Everolimus block cystine-induced cancer cell proliferation. In addition, cystine confers resistance to oxaliplatin and irinotecan chemotherapy by quenching chemotherapy-induced reactive oxygen species via synthesizing glutathione. We demonstrated that dietary deprivation of cystine suppressed colon cancer xenograft growth without weight loss in mice and boosted the antitumor effect of oxaliplatin. These findings indicate that cyst(e)ine, as part of supplemental nutrition, plays an important role in colorectal cancer and manipulation of cyst(e)ine content in nutritional formulations may optimize colorectal cancer patient survival.

## Introduction

Malnutrition is a common problem in colorectal cancer (CRC) patients, owing to clinical factors such as intestinal obstruction and cancer cachexia;^1^ thus parenteral and enteral nutrition (PEN) play important roles in the supportive care of these patients.^2,3^ Enteral nutrition (EN) generally refers to any method of feeding that uses the gastrointestinal (GI) tract to deliver part or all of the nutrients required for physiological functions in the body and EN should be preferred if the intestinal functions are preserved.^4^ Parenteral nutrition (PN), supplemental or total, is a route of nutrient administration that bypasses the GI tract and nutrient solutions or emulsions are infused into a vein for patients who cannot receive feedings enterally at all or in sufficient quantities.^5^

As metabolism in cancer cells is drastically different from that of non-malignant cells of the same tissue origin,^6^ the nutrient needs of cells in tumors are different from those of cells in normal tissues,^7^ implying that nutrition support for cancer patients should be different from patients with non-malignant diseases. However, clinical guidelines for nutrition support in cancer patients published by the European Society for Parenteral and Enteral Nutrition and the Chinese Society for Parenteral and Enteral Nutrition have not yet provided a comprehensive and detailed nutrition support scheme. The primary recommendation is that macronutrients in nutrient formulations for cancer patients should be of low carbohydrate, high fat, high protein, and high calorie.^8^ The impact of specific components of PEN such as amino acids on cancer progression has not been fully studied.

A number of studies suggest that dietary intervention on specific amino acid can affect cancer development and therapeutic effect. Meadows et al.^9^ and Fu et al.^10^ reported that concomitant dietary tyrosine-phenylalanine restriction decreased melanoma growth and enhanced the antitumor effect of chemotherapy against melanoma. Muthusamy et al.^11^ showed that dietary restriction of serine and glycine constrain tumor growth. Liu et al.^12^ revealed that a methionine and cystine double-deprivation diet suppressed glioma growth. Dietary restriction of methionine to 0.12% synergized with 5-fluorouracil (5-FU) and radiotherapy, to inhibit colon cancer patient-derived xenografts.^13^ Therefore, manipulation of specific amino acid contents in nutrition formulations may influence cancer outcomes.

Cancer cells highly depend on cystine, which may serve as a metabolic vulnerability target for cancer treatment.^14^ System X_C_^−^ (SLC7A11) is a cystine/glutamate antiporter by which tumor cells take up extracellular cysteine, in the form of cystine.^15^ Cysteine is critical for maintaining protein synthesis and redox homeostasis in tumor cells.^16^ In addition, cystine deprivation (CD) results in lethal lipid reactive oxygen species (ROS) accumulation and induces an iron-dependent cell death called ferroptosis.^17^ Badgley et al.^14^ showed that depletion of cyst(e)ine induced ferroptosis in Kras/p53-mutant pancreatic tumors. Cramer et al.^18^ generated an engineered cyst(e)inase enzyme that induced sustained depletion of the extracellular l-cyst(e)ine pool in animals when injected. Several studies demonstrated that cyst(e)inase inhibited cancer growth and improved survival in several cancer mouse models.^14,18,19^ Recently, Kshattry et al.^20^ reported that cyst(e)inase in combination with auranofin, a thioredoxin reductase inhibitor, had a synergistic antitumor effect on resistant pancreatic cancer xenografts.

We previously performed a retrospective cohort study^21^ to analyze PN-related factors and their association with cancer patient survival. In the current study, we report the association of quantities of specific amino acids in PN formulations with overall survival (OS) of patients with GI cancer. We also performed in vitro and in vivo experiments to examine the influence of dietary manipulation of cyst(e)ine on colon cancer growth and response to chemotherapy, and the molecular mechanisms involved in the impact of this amino acid on cancer biology. We found that PN-related cysteine component was associated with poor survival in patients with GI cancer. CD diet inhibited colon cancer growth in vivo and enhanced the response of colon cancer xenografts to oxaliplatin. These findings have potential clinical impact on colon cancer treatment.

## Results

### Parenteral nutritional component cysteine is associated with reduced OS in patients with GI cancer

**Fig. 1:**
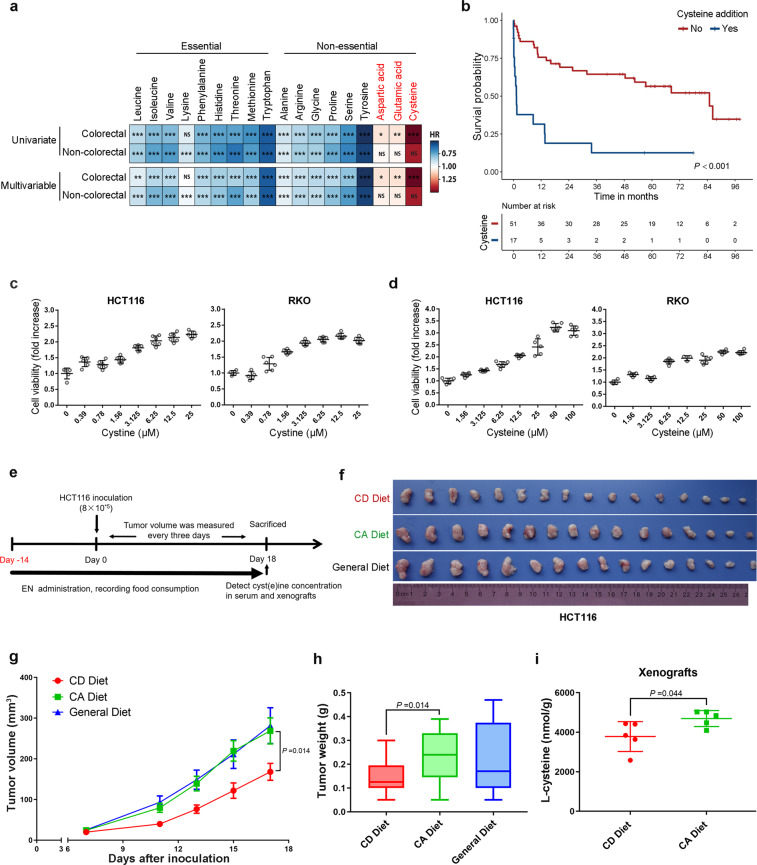
Dietary cystine addition promotes colon cancer growth and negatively correlates with patients’ clinical outcome. **a** Univariate and multivariable Cox proportional hazards analysis for the association of individual amino acids in PN formulations with OS in patients with colorectal cancer or other gastrointestinal cancers. Red color represents hazard ratio (HR) > 1.00 (poor OS), blue represents HR < 1.00 (good OS). NS indicates *P* ≥ 0.05, *P < 0.05, **P < 0.01, ***P < 0.001. **b** Upper panel: Kaplan–Meier curves showing OS for controls (patients with no PN cysteine, red) and cases (patients with PN cysteine, blue). *P*-value was determined by the log-rank test. Lower panel: patients-at-risk table for various time points. **c**, **d** Cyst(e)ine specifically promoted colon cancer cell growth in vitro. HCT116 and RKO colon cancer cells were cultured for 48 h in conditional media, containing gradient concentrations of cystine (0–25 μM) (**c**) or Cysteine (0–100 μM) (**d**). Cell viability was assessed by SRB assay. **e** Diagram shows the experimental protocol for enteral nutrition (EN) support and HCT116 colon cancer xenografting in BALB/c nude mice. **f**–**h** Cystine promoted colon cancer cell growth in vivo. Mice were subjected to CD (cystine deprivation) or CA (cystine addition) amino acids mixture diet or general diet. Tumor specimens were collected from killed mice on day 18 after HCT116 inoculation. Statistical analysis of tumor volumes (**g**) and tumor weights (**h**) in different groups (*n* = 8/group). *P*-value was determined by one-way ANOVA. **i** Xenograft cysteine levels were significantly lower in mice fed with CD diet than those in mice fed with CA diet. Quantification was performed by ultrahigh-performance liquid chromatography-mass spectrometry/mass spectrometry target metabolomics (*n* = 5/group). *P*-value was determined by Student’s *t*-test. Data are shown as mean ± SD (**c**, **d**, **i**), mean ± SEM (**g**), or 5–95 percentile (**h**)

We first sought to determine the impact of PEN-related amino acid components on outcomes in GI cancer patients. In terms of amino acid components, PN formulations are more comprehensively and clearly defined than EN formulations. Thus, we collected PN component data using the institutional pharmacy database for 1378 consecutive patients with GI cancer who received PN support at The University of Texas MD Anderson Cancer Center between 2008 and 2013. Patient characteristics are summarized in Supplementary Table 1. Among our study patients, 36.4% had chemotherapy within 30 days of the PN treatment. Almost half of the patients (41%) had colorectal (colon, rectal, or cecum) cancer. Univariate and multivariable Cox regression analysis indicated that most amino acids that are usually found in PN formulations had beneficial effects on GI cancer patient OS; however, the addition of cysteine, aspartic acid, or glutamic acid in the PN formulation was associated with shorter OS among CRC patients (Fig. 1a). To further investigate the effect of cysteine on the OS of patients with GI cancer, we ran a 1 : 3 ratio case–control analysis. Cysteine was significantly (*P* < 0.001) associated with shorter OS (Fig. 1b). GI cancer patients who received PN containing cysteine had shorter OS than GI cancer patients who received PN without cysteine (hazard ratio 4.71, 95% confidence interval 2.36–9.40, *P* < 0.001).

To clarify the function of these amino acids in CRC, we first tested growth of colon cancer cells (HCT116 and RKO) cultured in single amino acid-free or re-supplement media. Increasing concentrations of cystine (0–25 μM) or cysteine (0–100 μM) significantly promoted colon cancer cell growth in vitro in a dose-dependent manner, as reflected by the increase in protein biomass measured by the sulforhodamine B (SRB) assay (Fig. 1c, d). However, addition of glutamate (0–1.6 mM) or aspartate (0–1.6 mM) to conditioned medium only slightly promoted colon cancer cell growth (Supplementary Fig. 1c, d), although GI cancer patients who received PN containing glutamate or aspartate showed shorter OS (Supplementary Fig. 1a, b). Consistently, the cystine/glutamate antiporter *SLC7A11* mRNA is highly expressed in colon cancer tissues compared to adjacent normal tissues from GEPIA data (http://gepia.cancer-pku.cn/) (Supplementary Fig. 1e). Then, we knocked down SLC7A11 by small interfering RNAs (siRNAs) (Supplementary Fig. 1f) and found that depletion of SLC7A11 reduced cystine-mediated cell growth in both colon cancer cell lines (Supplementary Fig. 1g). In addition, we demonstrated that pharmacological inhibition of cystine transporter system X_C_^−^ activity by sulfasalazine^22^ significantly reduced cystine-mediated HCT116 and RKO cell growth in a dose-dependent manner (Supplementary Fig. 1h), indicating that intracellular functional cyst(e)ine was mainly transported from extracellular cystine by system X_C_^−^. These results suggest that cystine specifically promotes colon cancer cell growth and cyst(e)ine component of PEN is a hazard factor for colon cancer patients.

### Cystine-containing diet promotes colon cancer growth in vivo

To further confirm the pro-proliferation role of cyst(e)ine in CRC in vivo, we designed two amino acid mixture diets of cystine deprivation (CD) or cystine addition (CA) for mice with equal energy and nitrogen (Supplementary Table 2). As the methionine cycle contributes to de novo cysteine synthesis^15^ and methionine has anti-aging properties,^23^ we restricted methionine^24^ to 0.3% (w/w) in diets, which is equivalent to about a 50% reduction of methionine compared to general mouse chow. Then, mice were subjected to CD/CA diet or general diet for 2 weeks and then inoculated with HCT116 cells. Xenograft volume and mouse weight were monitored during the whole course (Fig. 1e). The addition of 0.4% (w/w) cystine in diet dramatically promoted xenograft growth and increased tumor weights (Fig. 1f–h). In contrast, CD diet showed a potent inhibition effect on colon tumor growth. We confirmed that the cysteine levels in xenografts were significantly decreased when the mice were fed with CD diet (Fig. 1i). Importantly, CD diet could normally sustain mouse body weight gain (Supplementary Fig. 1i) and no mice died during the study. Moreover, mean food and protein consumption per mouse were similar between CD and CA diets (Supplementary Table 3), indicating that tumor growth promotion attributed to cystine but not food intake-related energy alteration. These results suggest that cystine indeed promotes colon cancer growth in vivo.

### Cystine promotes colon cancer cell cycle G1/S transition and DNA synthesis

**Fig. 2:**
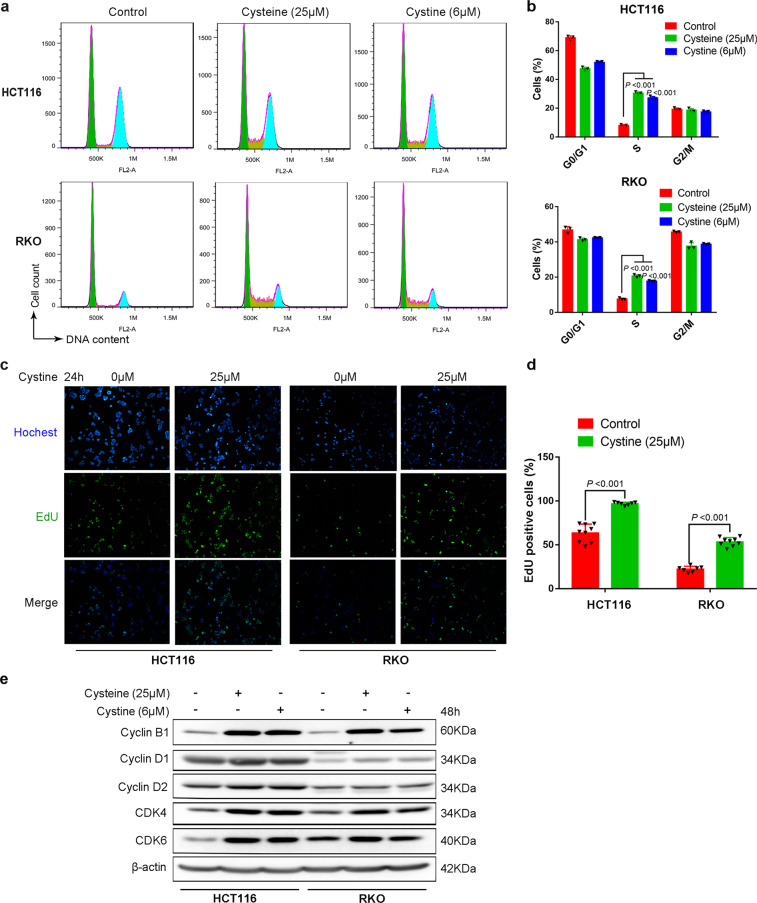
Cystine promotes colon cancer cell cycle progression and DNA synthesis. **a** Cystine promoted colon cancer G1/S cell cycle progression. HCT116 and RKO cells were cultured for 48 h in cyst(e)ine-free or 25 μM cysteine, 6 μM cystine-contained media, then cell cycle distribution was analyzed by flow cytometry and FlowJo software. Representative images are shown. **b** Quantitative results of **a**. *P*-value was determined by one-way analysis of variance. **c** Cystine promoted colon cancer cell DNA synthesis. HCT116 and RKO cells were cultured for 24 h in conditional media containing 0 μM or 25 μM cystine and DNA synthesis was assessed using the Click-iT EdU Alexa Fluor 488 Imaging Kit. The images were taken at ×100 magnification. Representative images are shown. **d** Quantitative results of **c**. *P*-value was determined by Student’s *t*-test. **e** Cystine increased the expression of CylinB1/D1/D2 and CDK4/6. Cell lysates were collected for WB analysis to detect cell cycle-related proteins. β-Actin was used as the loading control. Data are shown as mean ± SD from at least three independent biological replicates (**b**, **d**)

To determine whether cystine promotes colon cancer cell proliferation, we cultured HCT116 and RKO cancer cells in cystine-free or re-addition media and measured cell cycle by flow cytometry. As expected, we observed that cyst(e)ine promoted colon cancer cell cycle G1/S transition. The percentage of S-phase cells were significantly increased in the presence of cyst(e)ine (Fig. 2a, b). In line with this, cyst(e)ine significantly promoted DNA synthesis, as measured by the 5-Ethynyl-2′-deoxyuridine (EdU) incorporation assay (Fig. 2c, d and Supplementary Fig. 2b, c). In addition, cystine also increased the protein levels of cyclin B1/D1/D2 and cyclin-dependent kinase CDK4/6 (Fig. 2e). Furthermore, we performed RNA-sequencing (RNA-seq) and identified cystine-regulated genes (Supplementary Table 4). Gene Ontology (GO) enrichment analysis revealed that cystine regulated biological functions of G1/S transition of mitotic cell cycle and regulation of DNA replication biosynthetic process (Supplementary Fig. 2a). Therefore, we conclude that cystine promoted colon cancer cell proliferation mainly by promoting cell cycle G1/S transition and DNA synthesis.

### Cystine promotes colon cancer proliferation most likely by activating mTORC1

Next, we sought to determine the mechanism by which cystine promotes colon cancer cell proliferation. It is well known that cystine plays an important role for glutathione (GSH) production.^25^ We first tested whether cystine promoted colon cancer cell proliferation via increasing GSH synthesis. Although cellular total GSH was indeed significantly increased by cysteine (Supplementary Fig. 3a), blockade of GSH synthesis by l-Buthionine-sulfoximine (BSO) failed to suppress cystine-mediated colon cancer cell proliferation in both HCT116 and RKO (Supplementary Fig. 3b, c), indicating that GSH synthesis cannot explain cystine-mediated colon cancer cell proliferation.

**Fig. 3:**
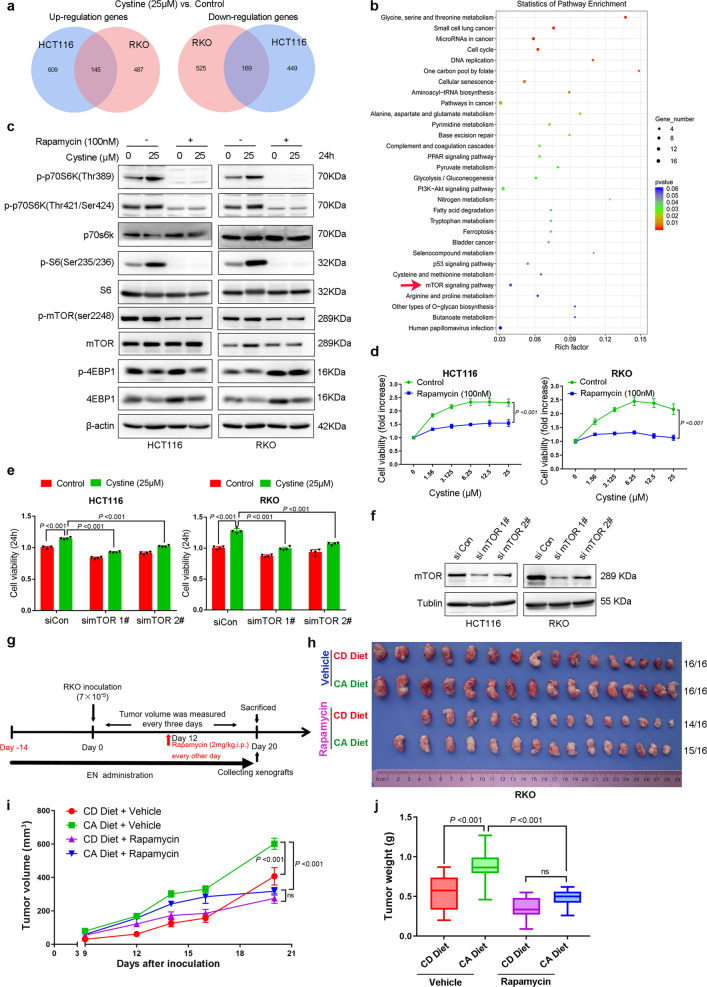
Cystine promotes colon cancer growth most likely by activating mTORC1. **a** Venn analysis was performed to identify genes co-regulated by cystine in HCT116 and RKO cells, including 145 co-upregulated and 169 co-downregulated genes. Data were extracted from RNA-seq results. **b** Cystine regulates mTOR signaling pathway according to the KEGG enrichment analysis in colon cancer. **c** Cystine activates mTORC1, as indicated by p-p70S6K/p70S6K, p-S6/S6 in HCT116, and RKO cells. Cells were cultured for 48 h in conditional media containing 0 μM or 25 μM cystine combined with 100 nM rapamycin. Cell lysates were collected for WB analysis. **d** mTORC1 inhibitors rapamycin blocked cystine-induced colon cancer cell growth. Cells were cultured for 48 h in conditional media with gradient concentrations of cystine (0–25 μM), alone or in combination with 100 nM rapamycin. Cell viability was detected by SRB assay. **e**, **f** Knockdown of mTOR rescued cystine-mediated cell growth. Cell culture media were replaced with conditional media containing 0 or 25 μM cystine after 36 h of transfection of siRNAs, and then the cells were continued to culture for 24 h and cell viability was detected by the SRB assay (**e**). mTOR was silenced by two siRNAs and were validated by WB analysis. Tublin was used as the loading control (**f**). **g** Diagram shows the experimental protocol for EN support, RKO colon cancer xenografting, and rapamycin injection (intraperitoneal) in BALB/c nude mice. **h** mTORC1 inhibitor rapamycin blocked cystine-induced colon tumor growth in vivo. Mice were subjected to a CD or CA diet, alone or in combination with DMSO or rapamycin injection. Tumor specimens were collected on day 20 after tumor inoculation. Both CD and CA diets contained 0.3% methionine. 14/16, 15/16, and 16/16 indicated that 16 points were inoculated and 14–16 xenografts were formed, as shown on the right. **i**, **j** Rapamycin blocked cystine-induced colon tumor growth in vivo. Statistical analysis of tumor volumes (**i**) and tumor weights (**j**) in different diet groups (*n* = 8/group). *P*-value was determined by one-way analysis of variance. Data are shown as mean ± SD (**d**, **e**), mean ± SEM (**i**), and 5–95 percentile (**j**)

According to the RNA-seq data in HCT116 and RKO cells, which were cultured for 48 h in cystine-free or 25 μM cystine-containing media, we found that cystine upregulated 145 genes and downregulated 169 genes in both HCT116 and RKO cells (Fig. 3a and Supplementary Table 4). Kyoto Encyclopedia of Genes and Genomes (KEGG) enrichment analysis showed that mammalian target of rapamycin (mTOR) signaling pathway, a well-known nutrient sensor, was regulated by cystine (Fig. 3b). Indeed, cystine activated mTORC1 activity, as determined by increased phosphorylation levels of p70 ribosomal protein S6 kinase (p70S6K) at both Thr389 and Thr421/Ser424 site, and S6 ribosomal protein (S6) (Fig. 3c and Supplementary Fig. 3e), whereas mTOR and eIF4E-binding protein 1 (4EBP1) protein phosphorylation levels were not affected by cystine. Interestingly, cystine dramatically decreased 4EBP1 protein levels (Fig. 3c). To validate whether cystine increases 4EBP1 phosphorylation, we stimulate colon cancer cells with cystine for 30 min and found that the phosphorylation levels of 4EBP1 were upregulated (Supplementary Fig. 3d). Subsequently, we treated colon cancer cells with the mTORC1 inhibitors rapamycin or everolimus and assessed cell viability. As expected, both mTORC1 inhibitors inhibited mTORC1 activity (Fig. 3c and Supplementary Fig. 3e) and significantly blocked cystine-induced cell proliferation in multiple colon cancer cell lines (Fig. 3d and Supplementary Fig. 3f, g). Similarly, knockdown of mTOR also dramatically blocked cell growth induced by cystine (Fig. 3e, f). Furthermore, we determined whether mTORC1 activation contributed to colon cancer growth in vivo in response to EN-related cystine, we conducted a RKO xenograft assay in BALB/c nude mice, as the schematic diagram shows (Fig. 3g). We confirmed that CA diet significantly promoted RKO xenograft growth (Fig. 3h), compared to CD diet. However, rapamycin treatment (intraperitoneal injection) significantly rescued the increases of tumor volumes and weights induced by cystine (Fig. 3h–j). Taken together, these findings indicate that nutrient cystine promotes colon cancer growth most likely by activating mTORC1, rather than synthesizing GSH.

### Cystine activates mTORC1 through GCN2-ATF4-SESN2 axis

**Fig. 4:**
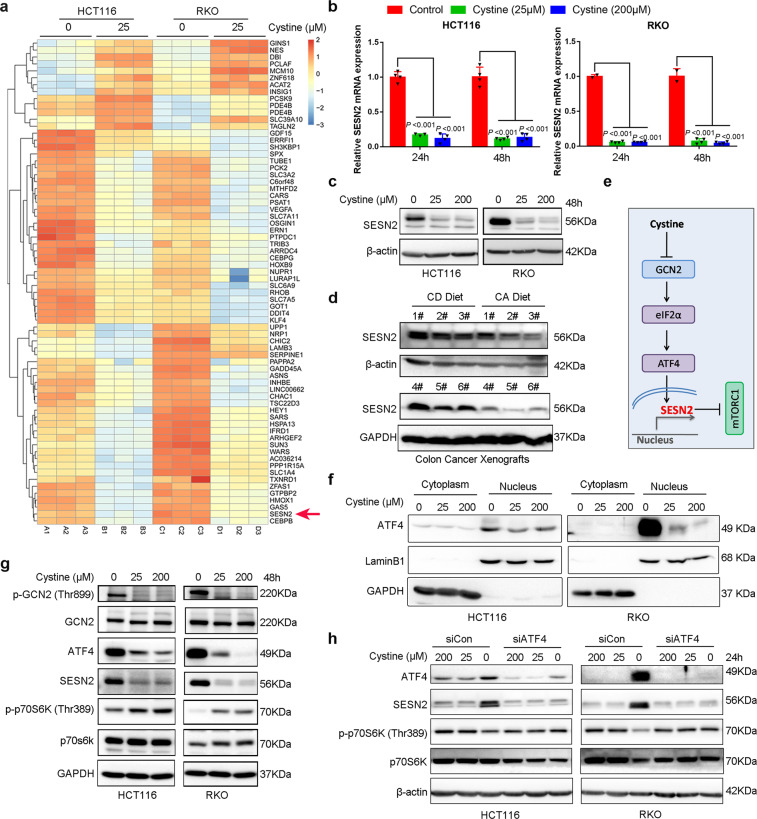
Cystine promotes mTORC1 activation via GCN2-ATF4-SESN2 axis. **a** Heatmap of genes that were significantly upregulated or downregulated by cystine in either HCT116 or RKO. *SESN2* was downregulated by cystine in both cell lines. **b** *SESN2* mRNA levels were dramatically downregulated by cystine in both HCT116 and RKO cell lines, as measured by quantitative real-time PCR. Cells were cultured for 24 or 48 h in conditional media with 0, 25, or 200 μM cystine. β-Actin was used as the loading control. *P*-value was determined by one-way analysis of variance. **c** SESN2 protein levels were dramatically downregulated by cystine in both HCT116 and RKO cell lines, as measured by WB. **d** SESN2 protein levels were downregulated by cystine in RKO xenografts, as shown in Fig. 3g. Six samples were analyzed by WB. **e** Schematic mechanism shows the mechanism by which cystine negatively regulates *SESN2* transcription and promotes mTORC1 activation. **f** Cystine reduced ATF4 level in nuclear fraction. Cells were cultured for 24 h in conditional media with 0, 25, or 200 μM cystine. Cell nuclear and cytoplasmic lysate were separated and subjected to WB analysis. **g** Cystine promotes mTORC1 activation via GCN2-ATF4-SESN2 axis. WB analysis was performed to detect p-GCN2/GCN2, ATF4, SESN2, and p-p70S6K/p70S6K protein expression in HCT116 and RKO cells cultured with conditioned media. **h** ATF4 depletion blocked cystine deprivation-induced mTORC1 inactivation. HCT116 and RKO cells were cultured for 24 h in conditional media with 200, 25, or 0 μM cystine and cell lysates were collected for WB analysis to detect protein expression. Data are shown as mean ± SD (**b**)

To illustrate the mechanism by which cystine activates mTORC1, we checked the genes that were significantly regulated by cystine (Fig. 4a and Supplementary Table 5) and noticed that *SESN2* was one of the most significantly downregulated genes by cystine in both cell lines. SESN2 is a highly conserved and stress-induced protein that inhibits mTORC1 activation through the GATOR complex.^26^ We examined *SESN2* mRNA and protein expression levels in HCT116 and RKO cells, and found that SESN2 was indeed dramatically downregulated by cystine at both mRNA and protein levels (Fig. 4b, c). Consistent with these findings, the SESN2 protein levels in RKO xenografts were downregulated by CA diet compared to CD diet (Fig. 4d).

Next, we investigated how cystine inhibits the mRNA expression of *SESN2*. When essential amino acids, including leucine, histidine, tryptophan, or lysine, are lacking, the eukaryotic initiation factor 2α (eIF2α) kinase general control nondepressible 2 (GCN2) in the brain and liver can be rapidly activated by binding to uncharged transfer RNAs, promoting eIF2α phosphorylation and inhibited translational initiation of most proteins.^27,28^ It has been reported that deprivation of glutamine, leucine, and arginine activated GCN2, increased the expression of transcription factor 4 (ATF4) to induce expression of SESN2, and inactivated mTORC1.^29^ We hypothesized that cystine activates mTORC1 also through the GCN2-ATF4-SESN2 axis in colon cancer cells. Indeed, cystine inhibited GCN2 phosphorylation and downregulated nuclear ATF4 protein levels (Fig. 4f), which decreased transcription of *SESN2* and ultimately activated mTORC1 in both HCT116 and RKO colon cancer cell lines (Fig. 4e–g). Consistently, CD increased the ATF4 and SESN2 protein expression and suppressed mTORC1 activation (Fig. 4g, h). Knockdown of ATF4 blocked CD-induced SESN2 expression and mTORC1 inactivation (Fig. 4h). These data indicate that cystine activates mTORC1 via the GCN2-ATF4-SESN2 axis in colon cancer cells. These data indicated that cystine activates mTORC1 via the GCN2-ATF4-SESN2 axis in colon cancer cells.

### Cystine induces colon cancer chemoresistance

**Fig. 5:**
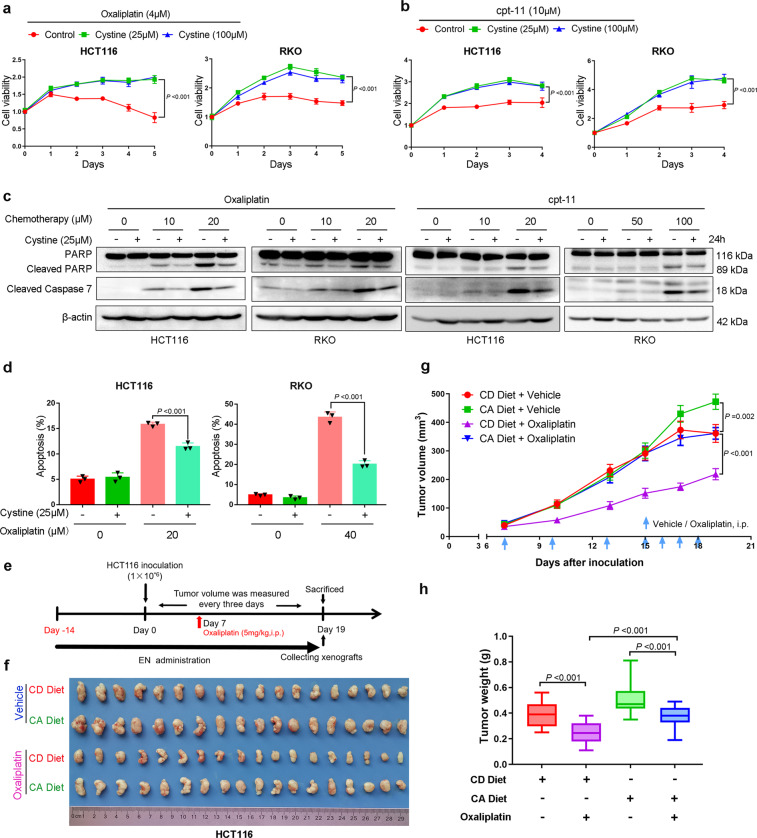
Cystine promotes colon cancer chemoresistance in vitro and in vivo. **a**, **b** Cystine promoted colon cancer resistance to oxaliplatin and irinotecan (cpt-11) in vitro. HCT116 and RKO cells were cultured with conditioned medium containing 0, 25, or 100 μM cystine and treated with 4 μM oxaliplatin (**a**) or 10 μM cpt-11 (**b**) for 2–5 days. Cell viability was detected by SRB assay. *P*-value was determined by one-way analysis of variance. **c** Cystine decreased colon cancer apoptosis in response to oxaliplatin and cpt-11 in vitro. HCT116 and RKO cells were cultured for 24–48 h in conditioned media with 0 or 25 μM cystine and treated with oxaliplatin (0–20 μM) or cpt-11 (0–100 μM). Cleaved caspase 7 and PARP expression were detected by WB analysis. **d** Cystine decreased colon cancer apoptosis in response to oxaliplatin in vitro. Apoptosis was detected by annexin V staining and flow cytometry analysis in HCT116 and RKO cells. The cells were cultured for 24 h in conditional media with 0 or 25 μM cystine, alone or in combination with 20 or 40 μM oxaliplatin. Quantitative results of three independent experiments were shown, *P*-value was determined by one-way analysis of variance. **e** Diagram shows the experimental protocol for EN support, HCT116 colon cancer xenografting, and oxaliplatin injection in BALB/c nude mice. i.p., intraperitoneal injection. **f** Mice were subjected to a CD or CA diet, alone or in combination with sterile water or oxaliplatin by intraperitoneal injection. Tumor specimens were collected on day 19 after tumor inoculation. **g**, **h** Cystine promoted colon cancer resistance to oxaliplatin in vivo. Statistical analysis of tumor volumes (**g**) and tumor weights (**h**) in different diet groups (*n* = 9/group). *P*-value was determined by one-way analysis of variance. Data are shown as mean ± standard deviation (**a**, **b**, **d**), mean ± SEM (**g**) and 5–95 percentile (**h**)

Oxaliplatin and irinotecan (cpt-11) are commonly used for colon cancer chemotherapy. We wondered whether cystine affects the response of colon cancer cells to chemotherapy. To answer this question, HCT116 and RKO cells were treated with oxaliplatin or cpt-11 under cystine-free or cystine-containing media. Cystine significantly induced chemoresistance to oxaliplatin and cpt-11 (Fig. 5a, b). In addition, oxaliplatin- and cpt-11-mediated DNA synthesis inhibition can also be partially blocked by cystine (Supplementary Fig. 4). Moreover, oxaliplatin- and cpt-11-induced apoptosis, as examined by Caspase 7 and poly (ADP-ribose) polymerase (PARP) cleavage (Fig. 5c) and annexin V staining (Fig. 5d and Supplementary Fig. 5a–c), were largely blocked by cystine in both cell lines. Finally, we performed HCT116 xenograft experiments and demonstrated that oxaliplatin (5 mg/kg) showed significant antitumor activity when the mice were subjected to CD diet (Fig. 5e–h). Consistent with previous results, xenografts grew faster and bigger in mice fed with CA diet. Most importantly, the antitumor efficacy of oxaliplatin in mice fed with CA diet was significantly compromised (Fig. 5e–h), as demonstrated that there was an average 38.4% reduction of tumor weight in CD diet group, whereas only a 26.2% reduction in CA diet after oxaliplatin therapy. These results suggest that cystine causes colon cancer chemoresistance in vitro and in vivo.

### Cystine promotes colon cancer chemoresistance via eliminating ROS by synthesizing GSH

**Fig. 6:**
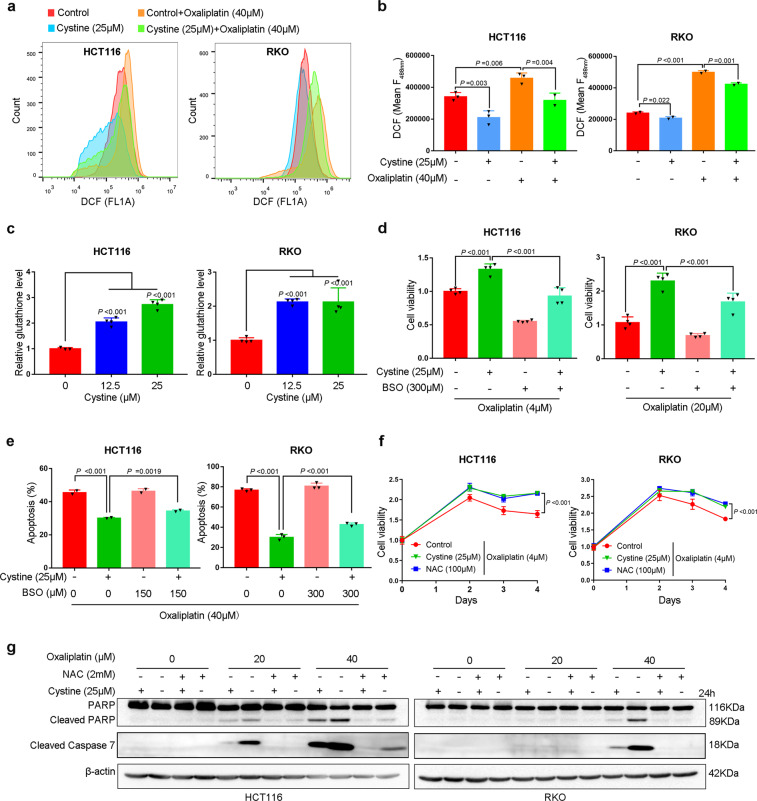
Cystine induces chemoresistance predominately by scavenging ROS via synthesizing GSH. **a** Cystine decreased ROS levels in colon cancer cells. Flow cytometry analysis of ROS levels using DCFDA staining in HCT116 and RKO cells. Cells were cultured for 24 h in conditioned media with 0 or 25 μM cystine, alone or in combination with 20 or 40 μM oxaliplatin. **b** Quantitative results of DCF using fluorescence intensity at 488 nm. *P*-value was determined by one-way analysis of variance. **c** Cystine significantly increased GSH levels in colon cancer cells. HCT116 and RKO cells were cultured for 12 h in conditional media with 0 or 25 μM cystine, *P*-value was determined by one-way analysis of variance. **d** Blockage of GSH synthesis by BSO abrogated cystine-induced oxaliplatin resistance. Cell viability were measured by the SRB assay after HCT116 and RKO cells were cultured for 96 h in conditional media with 0 or 25 μM cystine and treated with 4 or 20 μM oxaliplatin, alone or in combination with 300 μM BSO. *P*-value was determined by one-way analysis of variance. **e** BSO abrogated cystine-induced oxaliplatin resistance. Apoptosis was detected by annexin V staining and flow cytometry analysis. Cells were treated for 24 or 36 h with similar conditions from **d**. Quantitative results from three independent experiments were shown. *P*-value was determined by one-way analysis of variance. **f** NAC caused oxaliplatin resistance in colon cancer cells. Cell viability were measured by the SRB assay. Cells were cultured in conditioned media containing 0 or 25 μM cystine or 100 μM NAC, and treated with 4 μM oxaliplatin for 4 days. *P*-value was determined by one-way analysis of variance. **g** NAC and cystine decreased colon cancer apoptosis in response to oxaliplatin in vitro. Upon treatment with oxaliplatin (0–40 μM), HCT116 and RKO cells were cultured for 24 h in conditional media with 0 or 25 μM cystine or 2 mM NAC. Cleaved caspase 7 and PARP expression were detected by WB analysis. Data are shown as mean ± SD (**b**–**f**)

As cystine promoted cell growth by activating mTORC1, we sought to determine whether cystine-mediated chemoresistance is also through mTORC1. However, mTORC1 inhibitor rapamycin failed to rescue cystine-induced oxaliplatin resistance in both HCT116 and RKO cells (Supplementary Fig. 5d). Cytotoxic chemotherapy agents such as oxaliplatin and cpt-11 were reported to induce cellular ROS.^30^ In addition, hydrogen peroxide (H_2_O_2_)-mediated cell death was also partially rescued by cystine (Supplementary Fig. 5e); thereby, we speculated that cystine promoted GSH synthesis and scavenged excessive ROS, and then protects colon cancer cells from apoptosis. Indeed, we found that oxaliplatin and cpt-11 increased ROS production and cystine significantly reduced cellular ROS levels in HCT116 and RKO cells (Fig. 6a, b and Supplementary Fig. 6a, b). Accordingly, cystine increased GSH levels for more than twofold in both cell lines (Fig. 6c). When we used BSO to block cellular GSH synthesis (Supplementary Fig. 6c), cystine-mediated oxaliplatin and cpt-11 resistance in HCT116 and RKO cells were significantly rescued (Fig. 6d and Supplementary Fig. 6d). Oxaliplatin-induced apoptosis, as measured by annexin V staining, were also significantly restored (Fig. 6e and Supplementary Fig. 6e). In addition, the ROS scavenger *N*-acetylcysteine (NAC) entirely mimicked cystine to cause oxaliplatin and cpt-11 resistance (Fig. 6f and Supplementary Fig. 6f), and anti-apoptosis effect, as detected by the cleavage of Caspase 7 and PARP (Fig. 6g). Addition of GSH partially mimicked cystine to promote colon cancer resistance (Supplementary Fig. 6g). These findings suggest that dietary nutrient cystine induces chemotherapy resistance via anti-oxidation.

Finally, and most importantly, we also compared cystine with other nonessential amino acids including leucine, lysine, or arginine. As expected, compared to amino acids complete medium (AAs+), cell viability was attenuated when any one of these amino acid was deprived, but cystine or arginine deprivation showed the most significant inhibition effect (Supplementary Fig. 7a). However, surprisingly, upon oxaliplatin treatment, only CD increased colon cancer cell chemosensitivity; oxaliplatin-caused cell growth inhibition and oxaliplatin-induced apoptosis were further increased by deprivation of cystine rather than leucine, lysine, or arginine (Supplementary Fig. 7b–d), indicating that cyst(e)ine is more important in the progression of CRC.

## Discussion

Malnutrition is a common problem in GI cancer patients and PENs are commonly used for the nutrition support. To optimize the PEN formula, we must understand the impact of PEN-related specific components such as amino acids on cancer progression. Our retrospective analysis of GI cancer patients receiving PN, as well as further in vitro and in vivo studies, provided clinical evidence to confirm the corollary to the previous finding that cysteinase injection or dietary restriction of cyst(e)ine suppresses cancer—i.e., we found that nutritional supplementation with cysteine promotes cancer progression. Our findings suggest that CD in PEN should be considered for colon cancer patients.

Accumulating evidence suggests that cancer cells may addict to cystine and the cystine pathway could serve as a therapeutic target. Cysteine, a sulfur-containing nonessential amino acid, is widely used within cells for multiple processes, including catalysis, detoxication, metal trafficking, and response to oxidative stress.^31^ As system X_c_^−^ is responsible for importing cystine, several studies showed that suppression of SLC7A11 displayed antitumor effects.^32,33^ Correspondingly, we also confirmed that inhibition of SLC7A11 by siRNAs or sulfasalazine mostly rescued cystine-mediated cell growth (Supplementary Fig. 1f–h), although cystine still promoted cell growth (Supplementary Fig. 1) and sulfasalazine could further inhibit cell growth even in the absence of cystine (Supplementary Fig. 1h); this is probably because other transporters such as systems A, EAAT4/ASCT2 are also responsible for importing cyst(e)ine^34^ and sulfasalazine can also inhibit nuclear factor-κB signaling pathway^35^ besides targeting the system X_c_^−^. Cramer et al.^18^ reported that extracellular cyst(e)ine is necessary for growth and survival of several cancers.^14,18–20,36,37^ In addition, cyst(e)inase inhibited the growth of multiple types of tumors.^14,18,19^ Our studies fully support the notion that colon cancer cell growth and survival depend on the supply of cystine and CD is beneficial for colon cancer treatment in combination with chemotherapy.

In this study, we found that dietary intervention with CD obviously suppressed tumor growth without affecting mouse weight (Fig. 1f and Supplementary Fig. 1i). These results suggest that CD should be safe for colon cancer patients. Although our retrospective study suggest that cysteine deprivation PN are beneficial for GI cancer patients, prospective clinical trials will be required to test this hypothesis.

Cystine is the predominant form of cysteine extracellularly, because cysteine is rapidly oxidized to cystine in normoxic conditions,^25^ so we manipulated the levels of cystine in culture medium and diets during our study. Cysteine, in turn, is the prevailing form intracellularly owing to the highly reducing conditions^25^ and we detected cysteine levels in xenografts under CD diets. In addition to conversion from cystine directly, cysteine is mainly produced from methionine via the de novo transsulfuration pathway. A recent study showed that cysteine biosynthesis from methionine supported cancer cell growth upon extracellular cysteine limitation.^15^ In addition, the results of the cyst(e)inase study by Cramer et al.^18^ confirmed that extracellular cyst(e)ine is necessary in many cancers.^18–20,36^ Therefore, we restricted dietary methionine to a level equal to 50%^38^ reduction so that the cyst(e)ine levels in tumors can be maximally reduced but sufficient to maintain normal mouse weight gain.^24^

mTOR is an evolutionarily conserved serine/threonine protein kinase with two complexes, mTORC1 and mTORC2. mTORC1 is directly regulated by cellular energy and nutrient status such as amino acid levels.^39^ The two best-characterized downstream effectors of mTORC1 are p70S6K and 4EBP1, whose phosphorylation levels are commonly used as markers of mTORC1 activity.^40^ Cystine did not increase phosphorylation level of 4EBP1 at 24 h, possibly because cystine decreased 4EBP1 total protein levels (Fig. 3c). It has been reported that phosphorylated 4EBP1 will be ubiquitinated and degraded by proteasome.^41^ Indeed, we validated that cystine induced 4EBP1 phosphorylation at 30 min (Supplementary Fig. 3d). It was reported that several amino acids, including leucine, arginine, and glutamine, activate mTORC1 via different mechanisms.^42,43^ Activation of the mTORC1 by amino acids is associated with the translocation of mTORC1 to lysosomes, where it interacts with RHEB, a potent mTORC1 activator;^44^ thus, cystine did not affect mTOR protein phosphorylation level (Fig. 3c). SLC38A9 is a lysosomal arginine sensor.^42^ SESN2^45^ and CASTOR1^46^ are cytosolic leucine and arginine sensors, respectively. Amino acid stimuli can disrupt the interaction between their sensors and GATOR2, which activates mTORC1.^47^

Cystine promotes colon cancer cell proliferation and growth through activating mTORC1. mTORC1 inhibitors completely abrogated the pro-proliferation function of cystine in colon cancer cells (Fig. 3). To date, the mechanism behind how mTORC1 senses amino acids are complex and not yet fully understood. Wolfson et al.^45^ reported that leucine activated mTORC1 by binding to SESN2 directly and disrupting the SESN2–GATOR2 interaction. Ye et al.^29^ demonstrated that long-term starvation of leucine, arginine, or glutamine would result in a GCN2-dependent induction of SESN2 to maintain mTORC1 repression. For the first time, we proved that long-term starvation of cystine suppressed mTORC1 activity through the GCN2-ATF4-SESN2 axis in colon cancer cells.

Cystine promotes colon cancer cell chemoresistance through synthesizing GSH to eliminate ROS. Tumor resection in combination with 5-FU or capecitabine plus platinum-based chemotherapy has been widely used as the first-line therapy for colon cancer,^48^ and a major cause of recurrence and poor prognosis in CRC patients is chemotherapy failure. Thus, it is important to overcome chemoresistance in CRC patients. Nunes et al.^49^ reported that cysteine protected cells from carboplatin-induced death in ovarian cancer. We noticed that CD diet synergized with oxaliplatin to suppress colon cancer growth in vivo (Fig. 5). The possible mechanism is that cystine, similar to NAC and GSH, scavenges excessive ROS induced by cytotoxic drugs oxaliplatin (Fig. 6 and Supplementary Fig. 6). Although our experiments indicated only CD increased colon cancer cell sensitivity to oxaliplatin (Supplementary Fig. 7), the result may not fully explain the clinical association between PN with cystine and poor OS. Cystine is involved in many metabolic processes. Addition of cystine in PN may result in a wide range of metabolic disturbances that may benefit cancer cells and/or disrupt normal homeostasis.

Currently, cancer immunotherapy including anti-PD-1/PD-L1 restores or enhances the effector function of CD8^+^ T cells in the tumor microenvironment. A recent study showed that blocking glutamine metabolism induced a divergent metabolic program between effector T cells and cancer cells to overcome tumor immune evasion.^50^ Similarly, Wang et al.^51^ reported that the combination of cystine depletion by cyst(e)inase and PD-L1 blockade synergistically enhanced T-cell-mediated antitumor immunity in melanoma in vivo. Therefore, a logical next step is to determine whether dietary CD can synergize with anti-PD-1/PD-L1 therapy; this will form the basis of our future study plans.

In summary, our findings provided evidence that PEN-related cyst(e)ine promoted colon cancer growth through activating mTORC1. In addition, cyst(e)ine also promoted chemoresistance via synthesizing GSH to eliminate ROS. GI cancer patients with cystine-containing PEN showed poor survival (Supplementary Fig. 8). These discoveries suggest that cysteine-deprived PEN seems to be beneficial for the recovery of CRC patients.

## Materials and methods

### Clinical retrospective study and analysis

MD Anderson institutional pharmacy and tumor registry databases were used to identify all consecutive patients with GI cancer, who received PN support at MD Anderson Cancer Center between 1 August 2008 and 1 August 2013. The study was approved by the institutional review board of MD Anderson, which granted waivers of informed consent. Demographic, cancer-related, nutritional, and clinical data were collected. PN-specific data (i.e., dates of PN infusion, duration of PN support, and quantities of dextrose, fat, and specific amino acid components) were collected or calculated from pharmacy records. We defined OS as time from the date of first PN infusion to the date of death. Patients who were alive at the time of data abstraction were censored at the last known clinical contact date. The associations between PN-related amino acid parameters and OS of GI cancer patients were analyzed using univariate Cox proportional hazards regression analysis, followed by multivariable Cox proportional hazards regression analysis controlling for age, sex, race/ethnicity, Charlson comorbidity index, calorie-to-amino acid ratio, and non-PN calories, reporting the hazard ratios and *P*-values as a heatmap. To further investigate the association of the addition of cysteine in PN with outcomes of GI patients, we conducted a 1 : 3 ratio case–control subanalysis. Each case (a GI patient who received PN containing cysteine) was matched with three controls (1 : 3 ratio) using a propensity score. The propensity score was obtained using logistic regression for cysteine presence with other patient characteristics affecting OS, including the following: (1) PN first administration date, (2) age at first PN administration, (3) sex, (4) race/ethnicity, (5) type of cancer, (6) surgery, (7) Charlson comorbidity index, and (8) body mass index. Propensity score matching was used to obtain matched 1 : 3 samples of patients who had cysteine or not. An ad hoc check was done after matching, to confirm the balance of patient characteristics between the two groups using a two-sample *t*-test for continuous variables, Fisher’s exact test for binary variables, and Cochran–Mantel–Haenszel test for ordinal variables. Kaplan–Meier analysis followed by log-rank test was used to compare the OS distributions between the two groups. Cox proportional hazards regression analysis was used to investigate the association between cysteine and OS, reporting the hazard ratio and 95% confidence interval.

### Cell culture and conditioned media

Human CRC cell lines HCT116, RKO, SW620, and HT29 were cultured in high-glucose Dulbecco’s modified Eagle medium (DMEM; 4.5 mg/L glucose, 4 mM glutamine; Gibco) supplemented with 5% fetal bovine serum (FBS). All cell lines were cultured at 37 °C and 5% CO_2_, and were regularly tested for mycoplasma contamination. The above cell lines were obtained from kmcellbank or BeNa Culture Collection and authenticated by short tandem repeat DNA profiling.

For the individual amino acid starvation and stimulation experiments, cells were subjected to conditioned medium with or without the indicated amino acid for 1–5 days. Basic conditioned medium of high-glucose DMEM without glutamine, cystine, and methionine (Cat#21013024, Gibco) supplemented with 5% FBS was reconstituted as follows: (1) Cyst(e)ine-free/re-addition media: added l-methionine to 0.2 mM and l-glutamine to 4 mM (normal concentrations of high-glucose DMEM media) defined as cyst(e)ine-free media, and then added indicated concentrations of l-cyst(e)ine to media as cyst(e)ine re-addition media; (2) glutamate or aspartate-free/re-addition media: added l-cystine to 0.2 mM, l-methionine to 0.2 mM, and l-glutamine to 1 mM defined as glutamate or aspartate-free media, and then added 1.6 mM glutamate or 1.6 mM aspartate as re-addition medias; (3) AAs+ media: added l-glutamine to 4 mM, l-methionine to 0.2 mM, and l-cystine to 0.2 mM (normal concentrations of high-glucose DMEM media) defined as AAs+ media.

Basic conditioned medium of high-glucose DMEM without l-Arginine, l-Glutamine, l-Lysine, and l-Methionine (Cat# DML04, Caisson Labs, USA) supplemented with 5% FBS was reconstituted as follows: (1) arginine-free (Arginine−) media: added l-glutamine to 4 mM, l-methionine to 0.2 mM, and l-Lysine to 0.8 mM defined as Arginine− media. (2) Lysine-free (Lysine−) media: added l-glutamine to 4 mM, l-methionine to 0.2 mM, and l-Arginine to 0.4 mM defined as Lysine− media. (3) Leucine-free (leucine−) media: basic conditioned medium of high-glucose DMEM without l-leucine (Cat# DML03, Caisson Labs, USA) supplemented with 5% FBS defined as leucine− media;

### Cell viability and proliferation assays

Cell viability was measured by SRB assays. Briefly, cells were seeded in 96-well plates and replaced with conditioned medium after adherence. Then, cells were cultured for the indicated time and fixed with 10% trichloroacetic acid at room temperature for 30 min, followed by incubation with 0.4% SRB (w/v) solution in 1% acetic acid for 20 min at room temperature. Finally, the SRB was dissolved with 10 mM unbuffered Tris base and the absorbance was measured at a wavelength of 530 nm on a plate reader (Bio Tek).

To detect the DNA synthesis of CRC cells, we used the Click-iT EdU Alexa Fluor 488 Imaging Kit (Cat#C10337, Invitrogen) according to the manufacturer’s protocol. Briefly, HCT116 or RKO cells were seeded on coverslips (BD Biosciences) with conditioned medium, alone or treated with oxaliplatin or irinotecan. After 24 h, the cells were incubated with 10 μM EdU in conditioned medium for 4 h, followed by fixing, permeabilizing, and staining. For each sample, ten random fields were observed using fluorescence microscopy, and the total number of cells and EdU-positive cells were counted.

### Cell cycle and apoptosis analysis

HCT116 and RKO cells were treated with cyst(e)ine-free or addition media for 48 h. Cells were digested, collected, and fixed with pre-cooling 75% ethanol at 4 °C overnight. The next day, cells were washed with phosphate-buffered saline twice and a total of 1 × 10^6^ cells were incubated with 100 μl of dyeing buffer (0.6% NP-40) containing 0.1 mg/ml propidium iodide (a DNA dye that stains all DNA) and 1 mg/ml RNase A for 30 min at 37 °C in the dark. Finally, the stained cells were analyzed by flow cytometry and data were analyzed by FlowJo software (BD Biosciences, V10.6.2).

HCT116 and RKO cells were cultured for 24 h in conditional media, alone or combined with BSO and oxaliplatin/irinotecan, and then apoptosis was calculated following the manufacturer’s protocol (Apoptosis Detection Kit, Cat#556547, BD Biosciences). Briefly, cells were simultaneously stained with Annexin V-fluorescein isothiocyanate and propidium iodide. Apoptosis was examined by flow cytometry and 20,000 events were counted in each sample. Data analysis was carried out by BD AccuriR C6 (BD Biosciences).

### Colon cancer xenograft experiments

Six-week-old male BALB/c nude mice were purchased from Hunan SJA Laboratory (Changsha, Hunan, China) and housed in flow cabinets under specific pathogen-free conditions. Animal feeding and experiments were approved by the animal ethics committee of Kunming Institute of Zoology, Chinese Academy of Sciences. After a short period of adaptation, mice were randomly subjected to CD/CA diet (Trophic Animal Feed High-tech Co., Ltd, China) or general diet for 14 days before inoculation (*n* = 8–10/group). Food and water were supplied ad libitum. Then, colon cancer cells (8 × 10^5^ HCT116 cells or 7 × 10^5^ RKO cells) were injected subcutaneously into both sides of the groin of each mouse to establish the CRC xenograft model. Tumor volumes and mouse weights were measured every 3 days. For rapamycin and oxaliplatin experiments, mice were randomly re-divided into two groups for each diet group when the tumor volume was close to 50 mm^3^. Rapamycin (2 mg/kg) and oxaliplatin (5 mg/kg) were administered by intraperitoneal injection. Rapamycin was dissolved in dimethyl sulfoxide and then diluted by 40% PEG-300 + 5% Tween-80 + 54% normal saline. Oxaliplatin was dissolved and diluted by sterile water. CA or CD diet, as described above, were designed according to previous studies^10,38^ and detailed ingredients are listed in Supplementary Table S2.

### Detection of cysteine concentration in xenografts

HCT116 xenografts were submitted to the Biotree (Shanghai, China) for analysis and cysteine levels in xenografts were quantified by ultrahigh-performance liquid chromatography-mass spectrometry/mass spectrometry target metabolomics.

### mRNA-seq and analysis

HCT116 or RKO cells were cultured for 48 h in conditional media containing 0/25 μM cystine, and three independent samples were collected by Trizol reagent and subjected to mRNA-seq by the LC Bio (Zhejiang, China) and data analysis. RNA-seq data are available in Gene Expression Omnibus repository (GSE157894). Venn analysis, GO enrichment, and KEGG enrichment analysis were performed as described by Lc-bio (https://www.lc-bio.cn/).

### Measurement of GSH and ROS levels

HCT116 and RKO cells were cultured for 12 h in cystine-free or addition media, alone or combined with oxaliplatin and BSO. Total cellular GSH levels were determined by GSH detection kit (Cat#S0053; Beyotime, China) according to the manufacturer’s protocol. The experiments were repeated three times and results were normalized with standard solution provided with the kit according to the instructions. For cellular ROS detection, cells were treated as indicated, and then digested and re-suspended in phosphate-buffered saline plus 5% FBS. Then, suspended cells were incubated with 10 μM DCFH-DA (Cat#S0033; Beyotime, China) for 20 min at 37 °C in the dark, excess DCFH-DA was removed by washing the cells with phosphate-buffered saline three times, and the mean intensity of DCF fluorescence in 20,000 cells was analyzed by flow cytometry using FlowJo software (V10.6.2, BD Biosciences).

### Cell transfection and western blot analysis

We used the Lipofectamine 2000 reagent (Invitrogen) to transfect siRNA. Sequences of siRNAs are listed in Supplementary Table 7. Western blot (WB) analysis was performed by standard techniques as described in our previous study. Xenografts and cell lysates were collected for WB analysis and β-actin was used as the loading control. Cell nuclear and cytoplasmic lysate were separated by NE-PER™ Kit (Cat#78833, Thermo Scientific). Information about all antibodies and reagents is listed in the Key Resources Supplementary Table 6.

### Quantitative real-time PCR

Total mRNA was isolated by TRIzol reagent (Invitrogen). A reverse-transcription assay was performed by iScript cDNA Synthesis Kit (Bio-Rad) to obtain the complementary DNA, and then SYBR Green Select Master Mix (Cat#4472908, Applied Biosystems) was used to quantify *SESN2* and *β-actin* mRNA expression on the ABI-7900HT System (Applied Biosystems). Primer sequences are listed in Supplementary Table 7.

### Statistical analysis

All graphs were created using GraphPad Prism software and statistical analyses were calculated using SPSS 17.0 or R software (version 3.6.3, The R Foundation, http://www.r-project.org). Comparisons between two independent groups were assessed by two-tailed Student’s *t*-test. One-way analysis of variance with least significant differences was used for multiple group comparisons.

## Supplementary information

Marked-supplementary Materials

Clean-supplementary Materials

Dataset 1 for Supplementary Table 4

Dataset 2 for Supplementary Table 5

## Data Availability

The data sets of the study are available from the corresponding authors upon reasonable request.
