# Supplementary material for: Cyst(e)ine in nutrition formulation promotes colon cancer growth and chemoresistance by activating mTORC1 and scavenging ROS
Source: Signal Transduct Target Ther. 2021 May 28;6:188. doi: 10.1038/s41392-021-00581-9 (PMC8160199; doi:10.1038/s41392-021-00581-9)
Supplement: Supplementary file 1 — Marked-supplementary Materials [file 41392_2021_581_MOESM1_ESM.docx]

Supplementary Materials for

Cyst(e)ine in Nutrition Formulation Promotes Colon Cancer Growth and Chemoresistance by Activating mTORC1 and Scavenging ROS

Jiao Wu^1#^, Sai-Ching Jim Yeung^2#^, Sicheng Liu^1#^, Aiham Qdaisat^2^, Dewei Jiang^3^, Wenli Liu^4^, Zhuo Cheng^3^, Wenjing Liu^3^, Haixia Wang^3^, Chuanyu Yang^3^, Lu Li^5^, Zhongmei Zhou^3^, Rong Liu^3^, Ceshi Chen^3, 6, 7*^, Runxiang Yang^1*^

Correspondence to: yrx_research@163.com or chenc@mail.kiz.ac.cn

**This PDF file includes:**

Figures. S1 to S8

Tables S1 to S3, S6 & S7

**
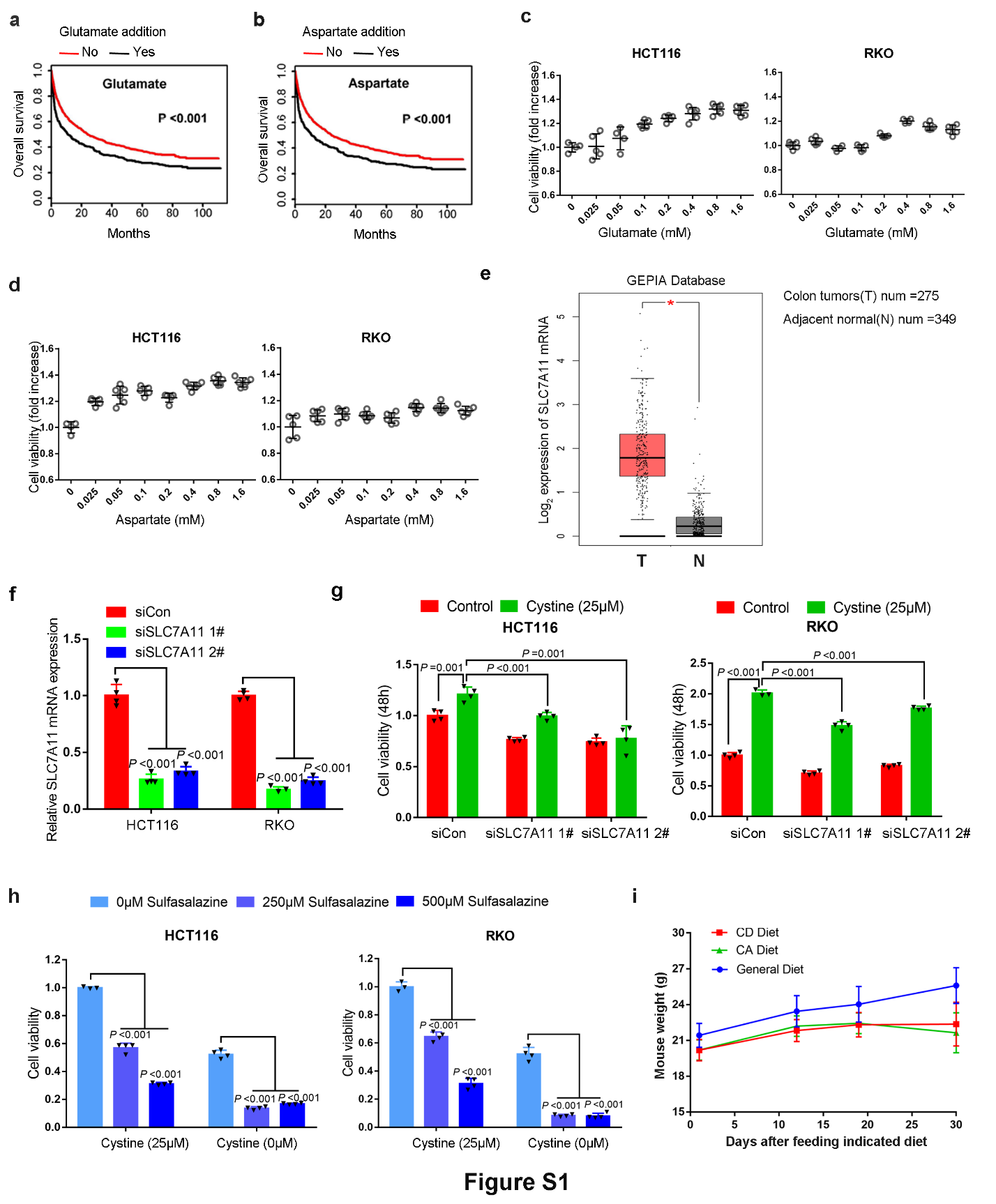
Supplementary Fig. 1** **Cystine but not glutamate or aspartate promotes cell growth of colon cancer cell lines. a, b** Aspartate and glutamate in PN were associated with poor survival of GI cancer patients. Kaplan-Meier curves are shown for indicated amino acid characteristics of PN. *P* value was determined by log-rank test. **c, d** Glutamate and aspartate did not consistently promote cell growth of colon cancer cell lines. Glutamate or aspartate only slightly promoted HCT116 and RKO cell growth. Cells were cultured for 48 hours in conditional media, containing gradient concentrations of glutamate (0-1.6mM) (**c**) and aspartate (0-1.6mM) (**d**), respectively. Then cell viability was detected by SRB assay. **e** *SLC7A11* mRNA expression levels in colon tumors are significantly higher than those in adjacent normal tissues from GEPIA data. **f** SLC7A11 was silenced by two siRNAs in HCT116 and RKO cells. The expression levels of SLC7A11 were validated by quantitative real-time PCR analysis. β-actin was used as the loading control**. g** Knockdown of SLC7A11 blocked cystine-mediated cell growth. Cell culture media were replaced with conditional media containing 0 μM or 25 μM cystine after 36 hours of siRNAs transfection, and then cells were continued to culture for 48 hours and cell viability was detected by the SRB assay. **h** SLC7A11 inhibitor sulfasalazine significantly decreased cystine-induced colon cancer cell growth. HCT116 and RKO colon cancer cells were cultured for 48 hours in the conditional media containing 0μM or 25μM cystine and treated with gradient concentrations of sulfasalazine (0-500μM). **i** Mouse weights were not affected by CD or CA diets. Statistical analysis of mouse weight gain in different groups (n= 8/group). *P* value was analyzed by Student’s *t*-test (**e**) and one-way analysis of variance (**f-h**). Data are shown as mean ± standard deviation from at least three independent biological replicates (**c-i**).

**
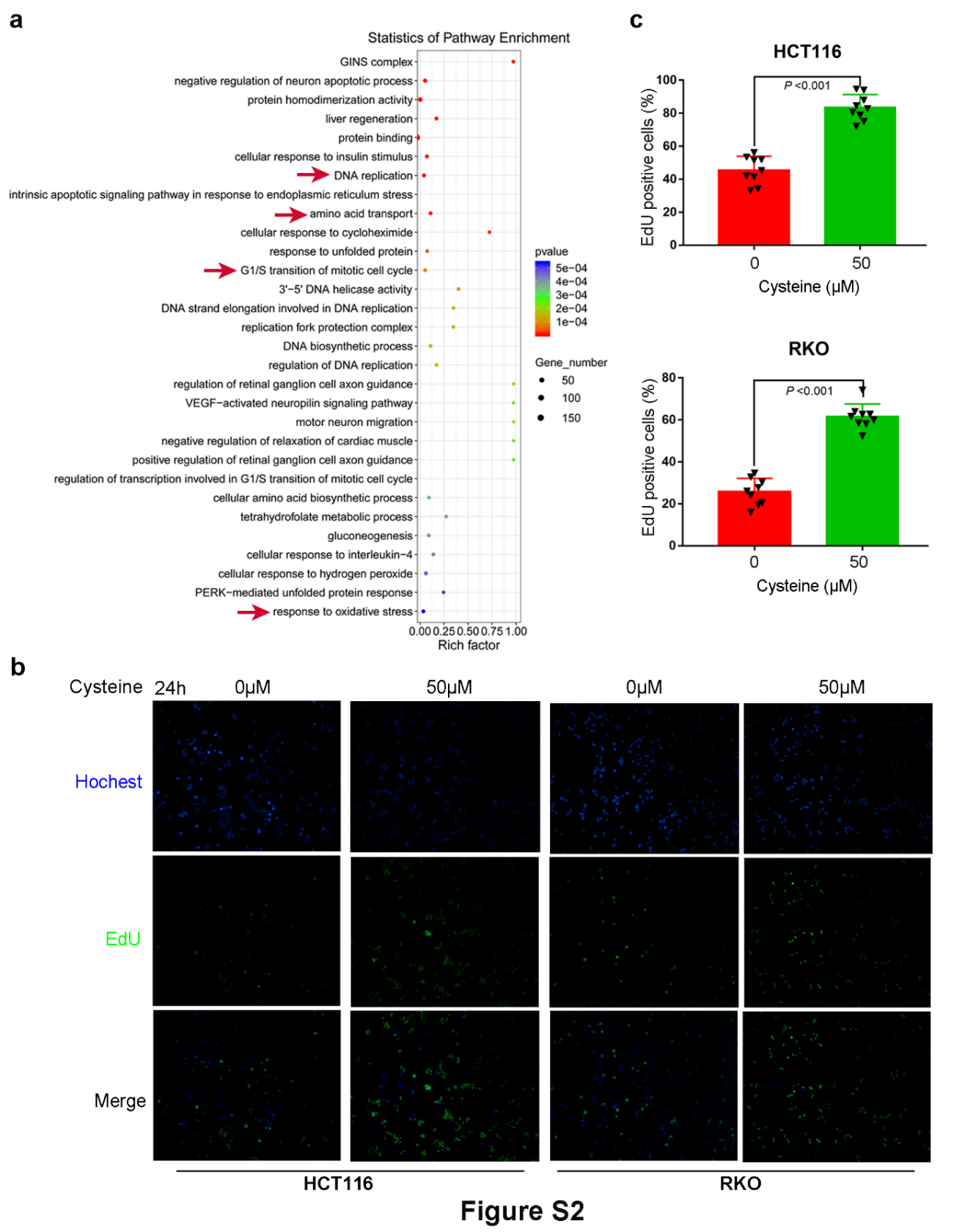
**

**Supplementary Fig. 2** **Cysteine promotes HCT116 and RKO colon cancer cell proliferation.** **a** GO enrichment analysis was conducted based on Veen results from Figure 3a to show cystine-related functions in colon cancer cells. **b** Cysteine significantly increased DNA synthesis of HCT116 and RKO cells. Cancer cells were cultured for 24 hours in conditional media with 0μM or 50μM cysteine, and DNA synthesis was assessed by the Click-iT EdU Alexa Fluor 488 Imaging Kit. Representative images are shown. **c** Quantitative results of (**b**). *P* value was analyzed by Student’s *t*-test (**c**). Data are shown as mean ± standard deviation (**c**).


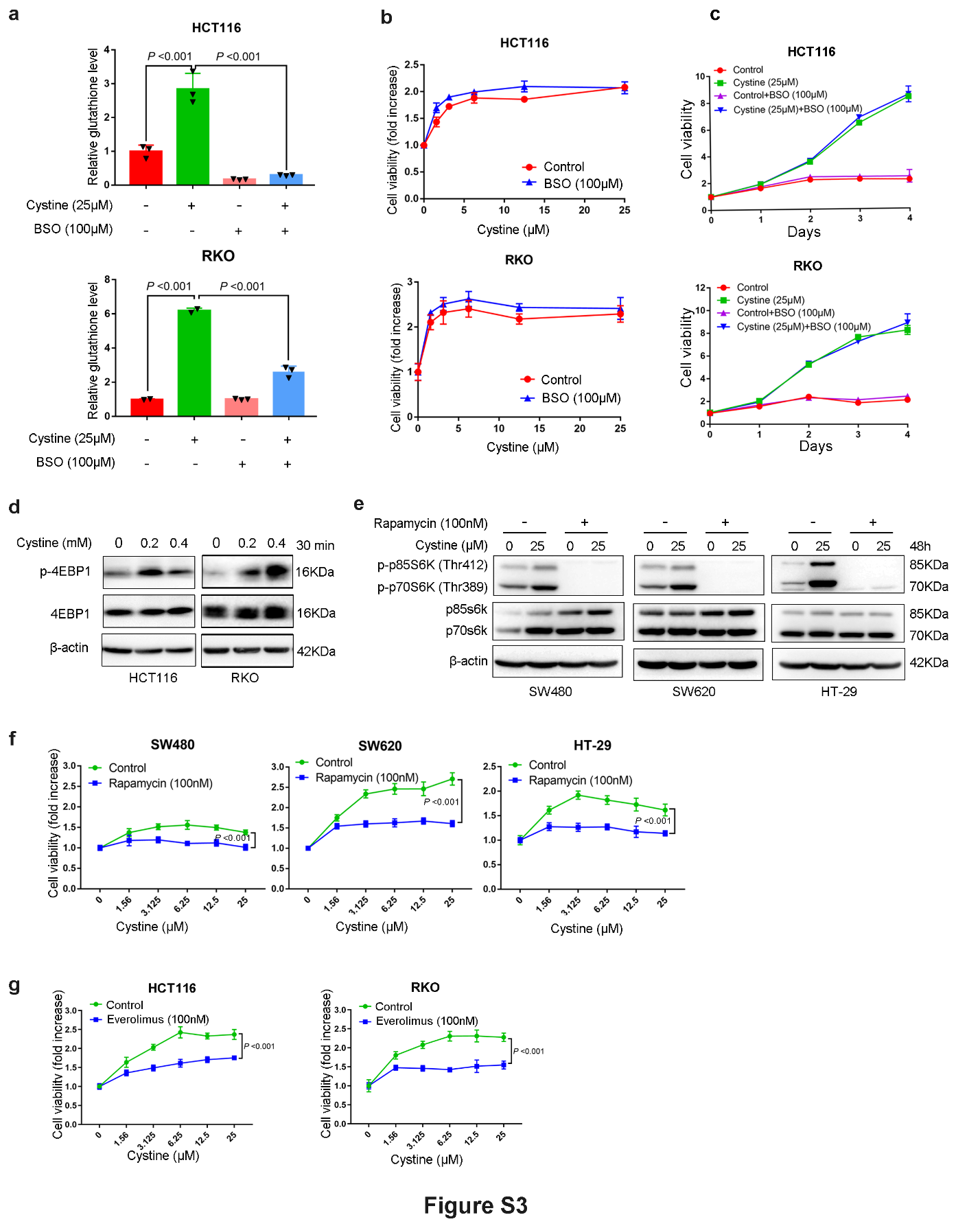
 **Supplementary Fig. 3**  **Inhibition of mTORC1 but not GSH prevents cystine-mediated cell growth in colon cancer cell lines.**

**a** BSO successfully blocked cystine-induced glutathione synthesis in HCT116 and RKO. Cells were cultured for 12 hours in conditional media containing 0μM or 25μM cystine combined with 100μM BSO treatment. **b, c** BSO did not block cystine-induced colon cancer cell growth. Cell viability was detected by the SRB assay. HCT116 and RKO cells were cultured for 48 hours in conditional media containing gradient concentrations of cystine (0-25μM) alone, or combined with 100μM BSO (**b**), or cultured in conditional media with 0μM or 25μM cystine alone, or combined with 100μM BSO for 4 days (**c**). **d** Short time stimulation of cystine increased phosphorylation level of 4EBP1. Cells were washed with 1×PBS for three times and incubated with Earle’s balanced salt solution contained 5% FBS for 2 hours at 37℃, then the cells were stimulated with indicated concentrations of cystine for 30 minutes. Cell lysates were collected for Western blot analysis. **e** Cystine activates mTORC1, as indicated by p-p70S6K/p70S6K inSW480, SW620 and HT-29 cells. Cells were cultured for 48 hours in conditional media containing 0μM or 25μM cystine alone, or combined with 100nM rapamycin. Cell lysates were collected for Western blot analysis. **f, g** mTORC1 inhibitors rapamycin and everolimus blocked cystine-induced colon cancer cell growth. Cells were cultured for 48 hours in conditional media with gradient concentrations of cystine (0-25 μM), alone or in combination with 100 nM rapamycin (**f**) or 100 nM everolimus (**g**). *P* value was analyzed by one-way analysis of variance (**a**) and Student’s *t*-test (**f, g**). Data are shown as mean ± standard deviation (**a-c, f and g**).

**
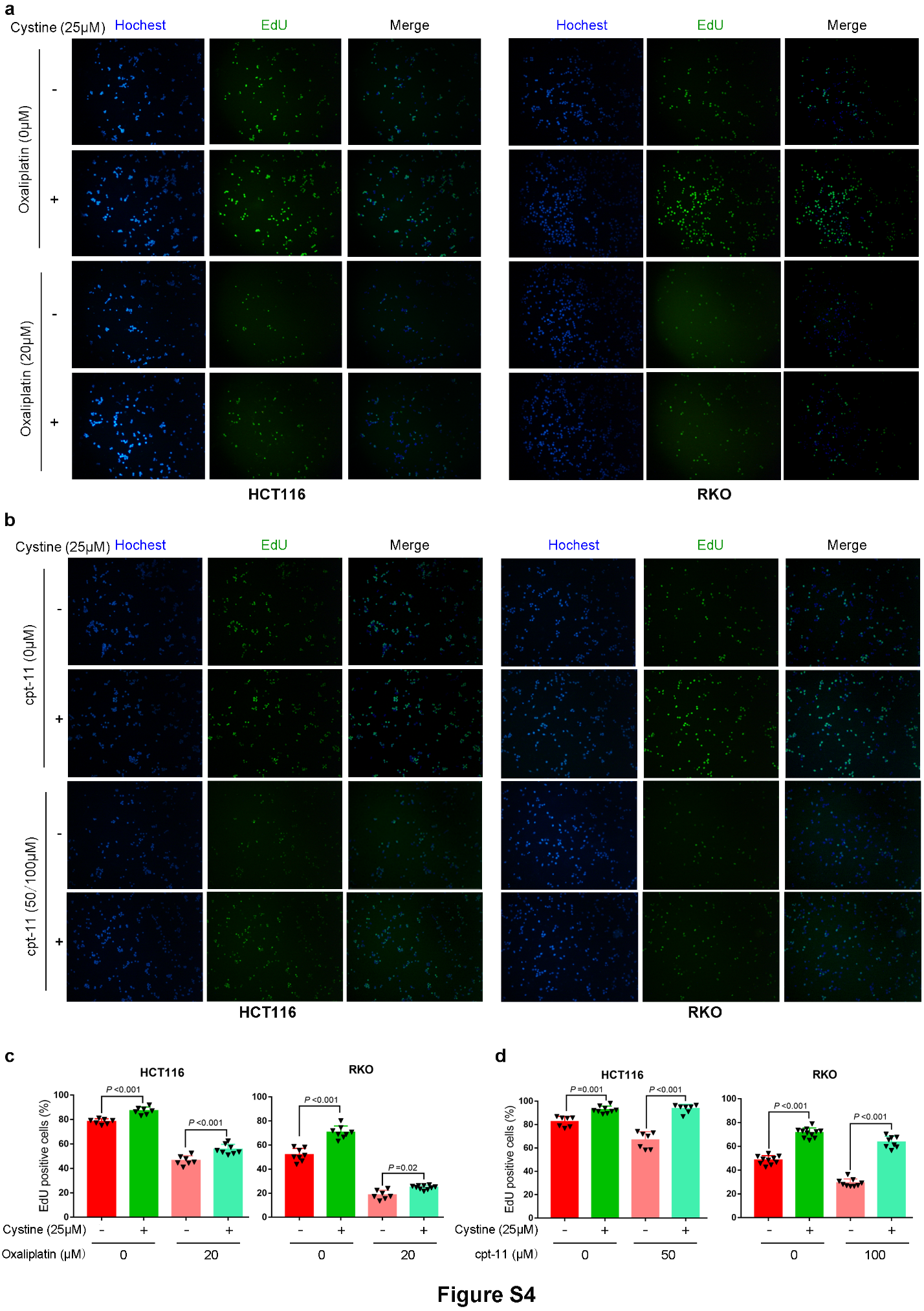
**

**Supplementary Fig. 4**  **Cystine rescued cell proliferative inhibition caused by oxaliplatin and irinotecan. a, b** HCT116 and RKO cells were cultured for 24 hours in conditional media containing 0 μM or 25 μM cystine alone, or combined with chemotherapy by 20 μM oxaliplatin (**a**) or 50/100 μM irinotecan (**b**), and DNA synthesis was assessed by the Click-iT EdU Alexa Fluor 488 Imaging Kit. Representative images are shown. **c, d** Quantitative results of oxaliplatin (**a**) and irinotecan (**b**) treatment are shown. *P* value was analyzed by one-way analysis of variance (**c, d**). Data are shown as mean ± standard deviation (**c, d**).


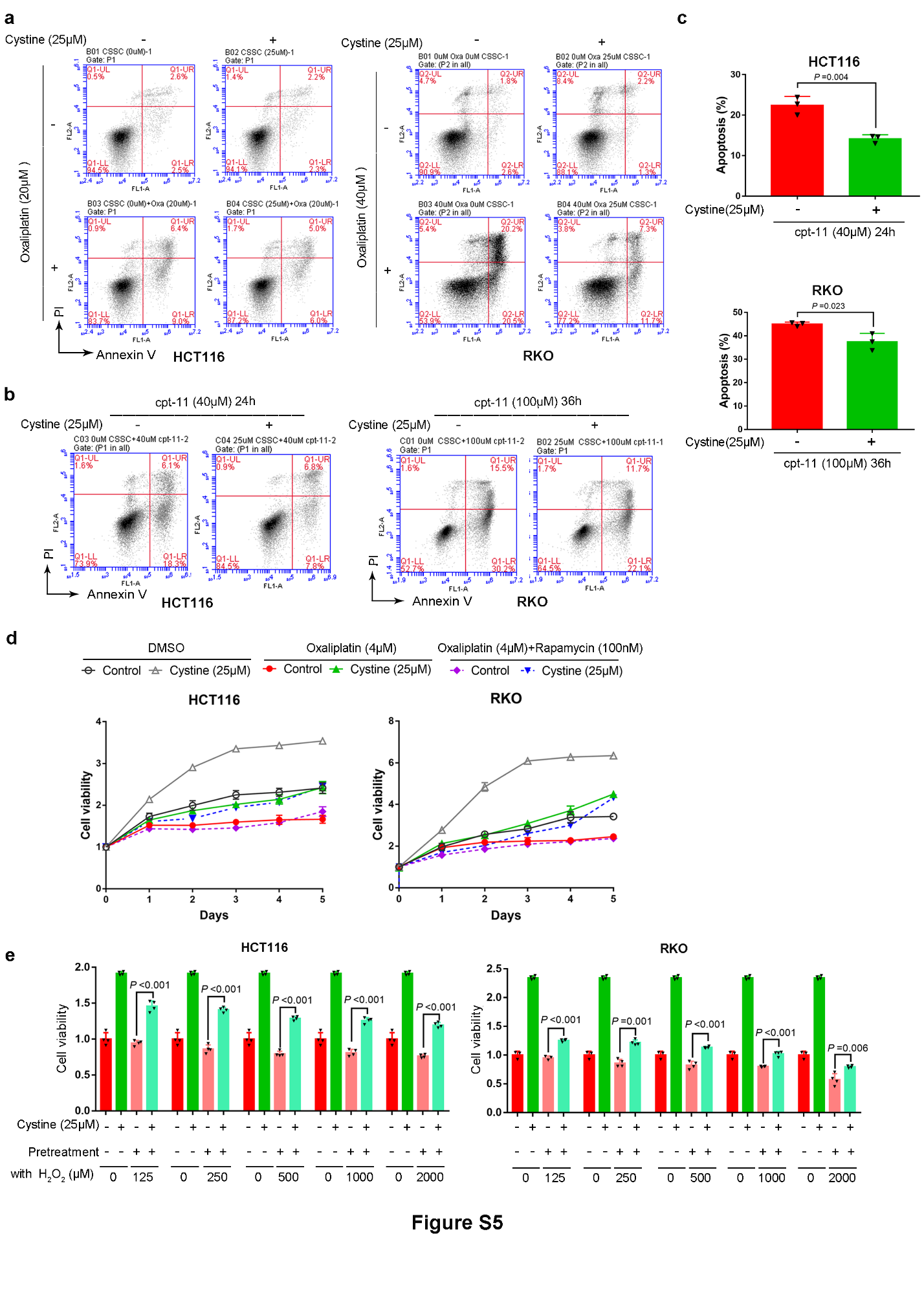


**Supplementary Fig. 5**  **Cystine decreased colon cancer apoptosis induced by oxaliplatin and irinotecan *in vitro*. a, b** Apoptosis was detected by annexin V staining and flow cytometry analysis in HCT116 and RKO cells. Cells were cultured for 24 hours in conditional media with 0 or 25 μM cystine, alone or in combination with 20/40 μM oxaliplatin (**a**) or 40/100 μM irinotecan (**b**). Representative images are shown. cpt-11, irinotecan. **c** Quantitative results of (**b**), and quantitative results of (**a**) was shown in Figure 5d. **d** Rapamycin failed to rescue cystine-mediated oxaliplatin resistance in HCT116 and RKO cells. Cancer cells were cultured in conditional media containing 0/25 μM cystine alone, or in combined with 4 μM oxaliplatin and 100 nM rapamycin for 5 days. **e** Cystine decreased hydrogen peroxide (H_2_O_2_) induced cell death. HCT116 and RKO cells were pretreated with indicated concentrations of hydrogen peroxide for 20 minutes, and then replaced with conditional media containing 0 or 25 μM cystine and cultured for 48 hours. Cell viability was detected by the SRB assay. *P* value was analyzed by Student’s *t*-test (**c, e**). Data are shown as mean ± standard deviation from at least three independent biological replicates (**c, d and e**).

**
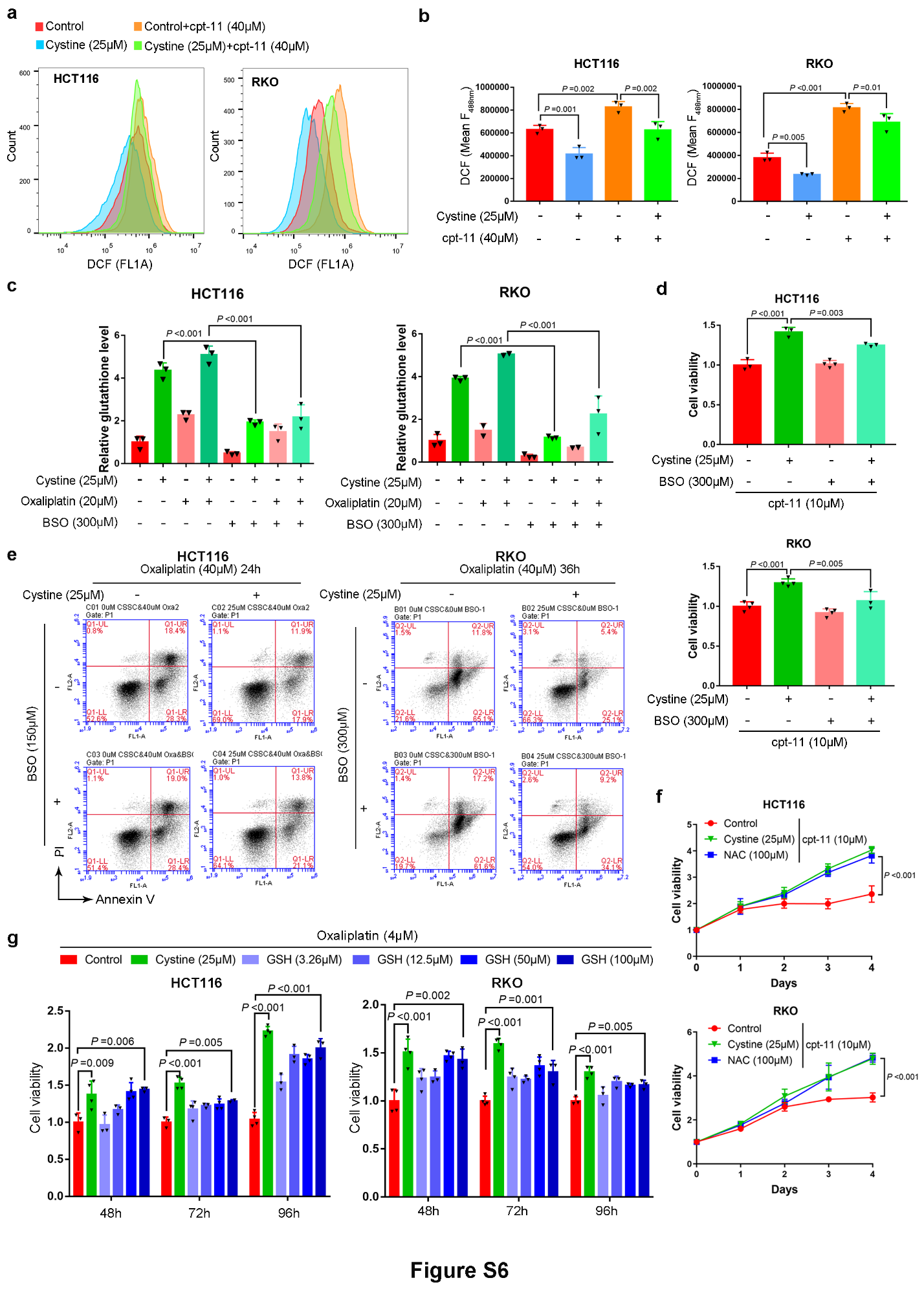
Supplementary Fig. 6**  **Cystine induces irinotecan-resistance also predominately by scavenging ROS. a, b** Cystine decreased irinotecan-induced ROS accumulation. Flow cytometry analysis of ROS levels using DCFDA staining in HCT116 and RKO cells. Cells were cultured for 24 hours in conditioned media with 0 or 25 μM cystine, alone or in combination with 40 μM irinotecan. Representative images are shown (**a**). Quantitative results of DCF using fluorescence intensity at 488 nm (**b**). **c** BSO blocked cystine-induced glutathione synthesis. Cells were cultured for 12 hours in conditional media alone, or combined with 20 μM oxaliplatin, 300 μM BSO, or both of them. Total glutathione levels were detected by GSH detection kit. **d** Blockage of GSH synthesis by BSO decreased cystine-induced irinotecan resistance. Upon 10 μM irinotecan treatment, the cells were cultured for 48 hours (RKO) or 72 hours (HCT116) in conditional media with 0/25 μM cystine, alone or in combination with 300 μM BSO. **e** BSO abrogated cystine-induced oxaliplatin resistance. Apoptosis was detected by annexin V staining and flow cytometry analysis in HCT116 and RKO cells. Representative images are shown. Quantitative results from three independent experiments were shown in Fig. 6e. **f** NAC caused irinotecan resistance in colon cancer cells. Cells were cultured in conditioned media containing 0/25 μM cystine or 100 μM NAC, and treated with 10 μM irinotecan for 4 days. **g** GSH partially mimicked cystine to promote colon cancer resistance to oxaliplatin. Cells were cultured in conditioned media containing 0/25 μM cystine or GSH (12.5/50/100 μM), and treated with 4 μM oxaliplatin for 2-4 days. Irinotecan, cpt-11. *P* value was analyzed by one-way analysis of variance (**b-d, f and g**). Data are shown as mean ± standard deviation (**b-d, f and g**).

**
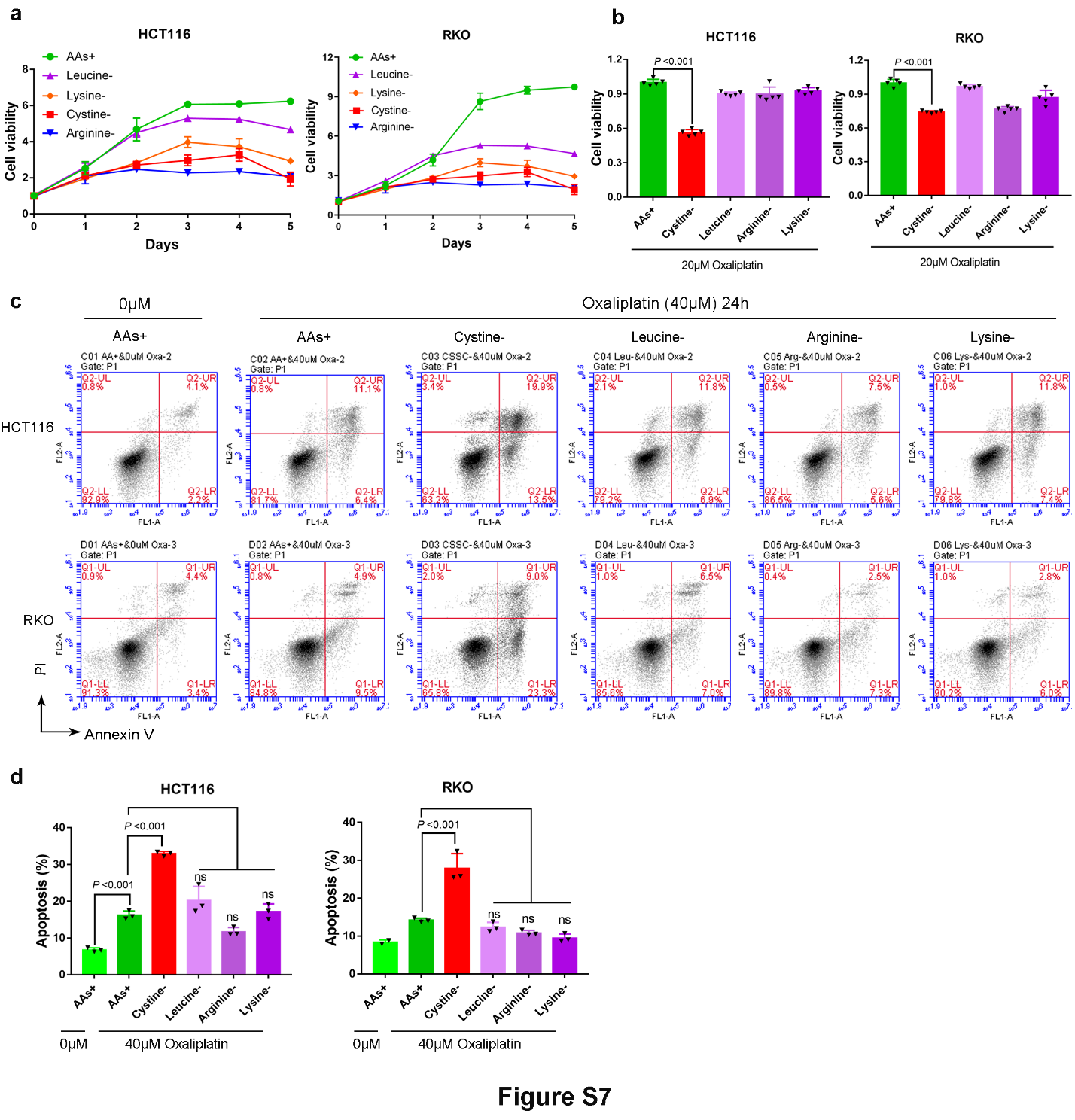
 Supplementary Fig. 7 Deprivation of cystine but not leucine, arginine or lysine increase chemosensitivity by scavenging ROS. a** Deprivation of cystine or arginine most significantly inhibited colon cancer cell growth. Cancer cells were cultured in amino acid complete media, or in conditional media with cystine, arginine, lysine or leucine deprivation, respectively for 5 days. Cell viability was detected by the SRB assay. **b, c** Cystine deprivation obviously increases colon cancer cell sensitivity to oxaliplatin. Upon 20 μM oxaliplatin treatment, cells were cultured in amino acid complete media, or in cystine, arginine, lysine or leucine deprivation conditional media. Then cell viability was detected by the SRB assay at 48 hours (**b**). Apoptosis was detected by annexin V staining and flow cytometry analysis at 24 hours, representative images (**c**) and quantitative results (**d**) are shown. *P* value was analyzed by one-way analysis of variance (**b, d**). Data are shown as mean ± standard deviation (**a-b, d**).
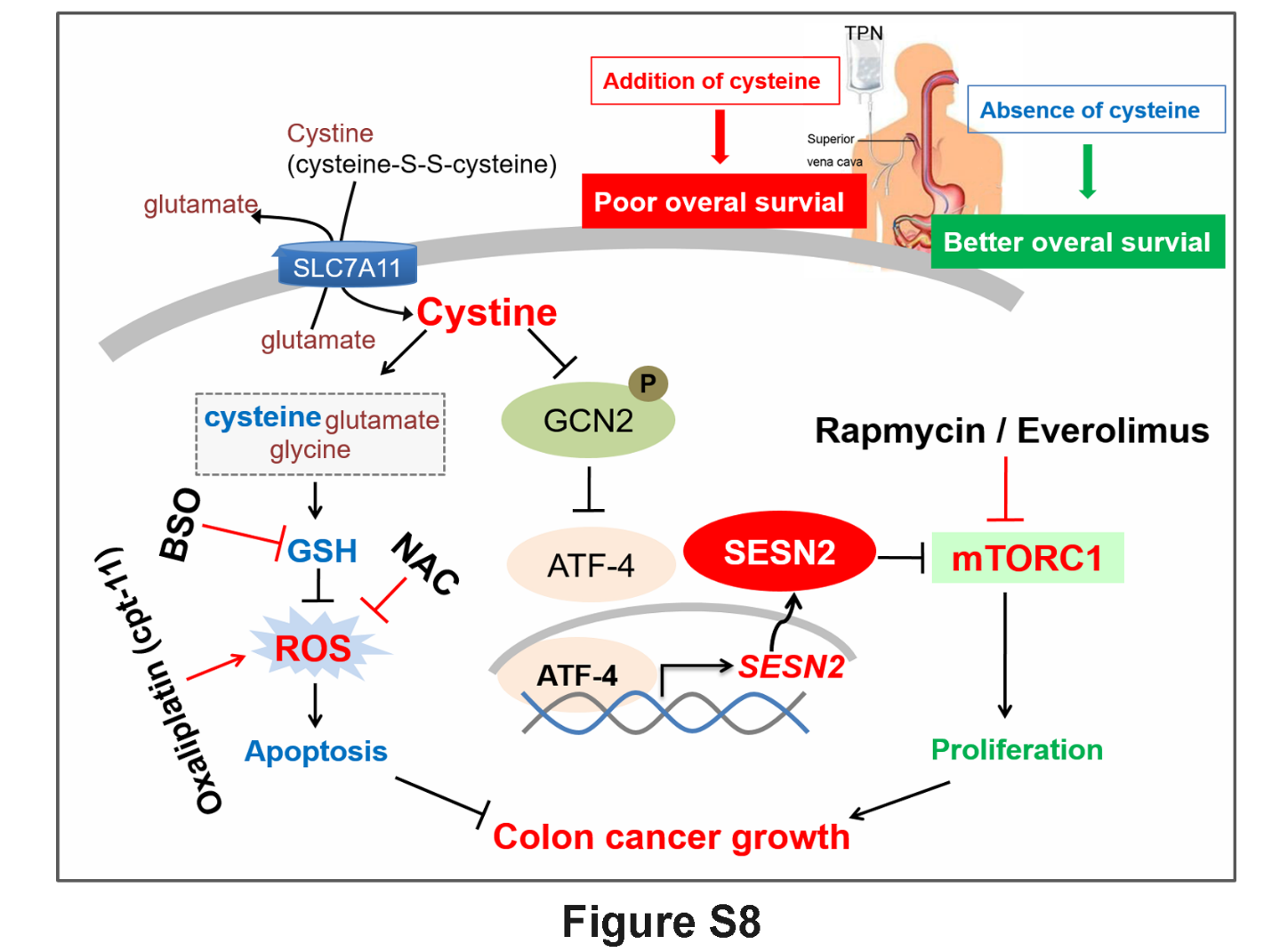
**Supplementary Fig. 8 Work model.** Summary for the roles of cyst(e)ine in nutrition formulation in promoting colon cancer growth and chemoresistance. GI cancer patients who received cysteine containing parenteral nutrition (PN) show poor survival than those who did not. Cystine promotes colon cancer cell growth in vitro and in vivo, predominately by inhibiting *SESN2* transcription via the GCN2-ATF4 axis, resulting in mTORC1 activation. mTORC1 inhibitors Rapamycin and Everolimus rescue cystine-induced cancer cell proliferation. Additionally, cystine confers resistance to oxaliplatin and irinotecan (cpt-11) chemotherapy by synthesizing GSH to eliminate chemotherapy-induced ROS, and the ROS scavenger N-acetylcysteine (NAC) entirely mimicked cystine to cause oxaliplatin resistance. When treated with BSO to block cellular GSH synthesis, cystine-mediated oxaliplatin resistance is significantly rescued.

**Supplementary Table 1. Characteristics of gastrointestinal cancer patients who received parenteral nutrition (PN).**

| **Characteristic** | **No. of patients (%)** |
| --- | --- |
| Total | 1378 |
| Mean age (SD) | 59 years (13 years) |
| Sex |  |
| Female | 595 (43.2) |
| Male | 783 (56.8) |
| Race/ethnicity |  |
| White | 969 (70.3) |
| Hispanic | 159 (11.5) |
| Black | 126 (9.1) |
| Others | 124 (9.0) |
| Gastrointestinal cancer subtype |  |
| Colon | 360 (26.1) |
| Pancreas | 213 (15.5) |
| Gastric | 171 (12.4) |
| Rectal | 160 (11.6) |
| Esophageal | 140 (10.2) |
| Small intestine | 97 (7.0) |
| Appendix | 80 (5.8) |
| Hepatobiliary | 52 (3.8) |
| Cecal | 45 (3.3) |
| Others | 60 (4.4) |
| Median body mass index (IQR) | 25.8 kg/m^2^(22.5-29.7 kg/m^2^) |
| Median Charlson comorbidity index (IQR) | 7 (6-8) |
| Surgery |  |
| No | 760 (55.2) |
| Yes | 618 (44.8) |
| Chemotherapy* |  |
| No | 876 (63.6) |
| Yes | 502 (36.4) |
| Mean daily PN calories (SD) | 20.7 kcal/kg (5.4 kcal/kg) |
| Mean daily PN amino acid content (SD) | 1.25 g/kg (0.28 g/kg) |
| Median treatment duration (IQR) | 10 days (6-17 days) |
| SD, standard deviation; IQR, interquartile range.  * Chemotherapy administration within 1 month prior to PN initiation and 1 month after PN completion. | |

| **Product** | **Cystine 0% + methionine 0.3% + NEAA 0.7%, (** **CD Diet )** | | **Cystine 0.4% + methionine 0.3% + NEAA 0.3%, (CA Diet)** | |
| --- | --- | --- | --- | --- |
|  | **gm** | **kcal** | **gm** | **kcal** |
| Content percentage |  |  |  |  |
| Protein | 17% | 17.5% | 17% | 17.5% |
| Carbohydrate | 68.5% | 70.8% | 68.5% | 70.8% |
| Fat | 5% | 11.6% | 5% | 11.6% |
| Total |  | 100% |  | 100% |
| Protein |  |  |  |  |
| L-arginine | 10.7 | 42.8 | 10.3 | 41.2 |
| L-histidine-HCl-H2O | 6.4 | 25.7 | 6.2 | 24.7 |
| L-isoleucine | 8.0 | 32.0 | 8.0 | 32.0 |
| L-leucine | 12.0 | 48.0 | 12.0 | 48.0 |
| L-lysine-HCl | 14.0 | 56.0 | 14.0 | 56.0 |
| L-methionine | 3.0 | 12.0 | 3.0 | 12.0 |
| L-phenylalanine | 8.0 | 32.0 | 8.0 | 32.0 |
| L-threonine | 8.0 | 32.0 | 8.0 | 32.0 |
| L-tryptophan | 2.0 | 8.0 | 2.0 | 8.0 |
| L-valine | 8.0 | 32.0 | 8.0 | 32.0 |
| L-alanine | 10.7 | 42.8 | 10.3 | 41.2 |
| L-asparagine-H_2_O | 5.4 | 21.4 | 5.2 | 20.6 |
| L-aspartate | 10.7 | 42.8 | 10.3 | 41.2 |
| **L-cystine** | **0.0** | 0.0 | **4.0** | 16.0 |
| L-glutamic acid | 32.1 | 128.4 | 30.9 | 123.6 |
| L-glutamine | 5.4 | 21.4 | 5.2 | 20.6 |
| Glycine | 10.7 | 42.8 | 10.3 | 41.2 |
| L-proline | 5.4 | 21.4 | 5.2 | 20.6 |
| L-serine | 5.4 | 21.4 | 5.2 | 20.6 |
| L-tyrosine | 4.3 | 17.1 | 4.1 | 16.5 |
| Carbohydrate |  |  |  |  |
| Corn starch | 550.5 | 2202.0 | 550.5 | 2202.0 |
| Maltodextrin 10 | 125.0 | 500.0 | 125.0 | 500.0 |
| Vitamin mix V10001 | 10.0 | 40.0 | 10.0 | 40.0 |
| Fat |  |  |  |  |
| Cellulose | 50.0 | 0.0 | 50.0 | 0.0 |
| Corn oil | 50.0 | 450.0 | 50.0 | 450.0 |
| Mineral mix S10001 | 35.0 | 0.0 | 35.0 | 0.0 |
| Choline bicarbonate | 7.5 | 0.0 | 7.5 | 0.0 |
| Choline tartrate | 2.0 | 0.0 | 2.0 | 0.0 |
| Total | 1000.0 | 3872.0 | 1000.0 | 3872.0 |
| NEAA, nonessential amino acid; Boldface, key manipulated amino acid. | | | | |

**Supplementary Table 2. Ingredients of enteral nutrition for mice.**

**Supplementary Table 3. Mean mouse weight gain and food consumption during the first 19 days of enteral nutrition administration.**

| **Diet** | **No. of mice** | **Mean weight gain during interval, g/mouse** | **Mean food consumption, g/day per mouse** | **Mean protein (amino acid) consumption, mg/g per mouse** | **Cystine consumption, mg/g** |
| --- | --- | --- | --- | --- | --- |
| CD Diet | 8 | 2.29 | 5.23 | 7376.56 | 0.00 |
| CA Diet | 8 | 2.24 | 4.61 | 6649.73 | 156.46 |
| General diet | 8 | 2.56 |  |  |  |
| CD, Cystine deprivation; CA, Cystine addition. | | | | | |

**Supplementary Table 6. Key Resources Table.**

| **Reagents and Resources** | **Source** | **Identifier** |
| --- | --- | --- |
| Antibodies |  |  |
| Anti-Phospho-p70 S6 Kinase (Thr389) (108D2) | Cell Signaling Technology | Cat#9234 |
| Anti- Phospho-p70 S6 Kinase (Thr421/Ser424) | Cell Signaling Technology | Cat#9204 |
| Anti-p70 S6 Kinase | Cell Signaling Technology | Cat#9202 |
| Anti-Phospho-S6 Ribosomal Protein (Ser235/236) | Cell Signaling Technology | Cat#4856 |
| Anti-S6 Ribosomal Protein (5G10) | Cell Signaling Technology | Cat#2217 |
| Anti-Phospho-mTOR (Ser2448) | Cell Signaling Technology | Cat#2971 |
| Anti-mTOR Antibody | Cell Signaling Technology | Cat#2972 |
| Anti-Phospho-4EBP1(Thr37/46) | ABclonal | Cat#AP0030 |
| Anti-4EBP1 | ZEN-Bio | Cat#306002 |
| Anti-Cleaved Caspase7 | Cell Signaling Technology | Cat#9492 |
| Anti-Cleaved PARP | Cell Signaling Technology | Cat#9541 |
| Anti-Cyclin B1 | Santa Cruz | Cat#SC-245 |
| Anti-Cyclin D2 (C-17) | Santa Cruz | Cat#SC-181 |
| Anti-Cyclin D1 (92G2) | Cell Signaling Technology | Cat#2978 |
| Anti-Cdk4 (H-303) | Santa Cruz | Cat#SC-749 |
| Anti-Cdk6 (B-10) | Santa Cruz | Cat#SC-7961 |
| Anti-GCN2 (phospho T899)[EPR2320Y] | Abcam | Cat#ab75836 |
| Anti-GCN2 | Cell Signaling Technology | Cat#3302 |
| Anti-ATF-4 (D4B8) | Cell Signaling Technology | Cat#11815 |
| Anti-Sestrin-2 (D1B6) | Cell Signaling Technology | Cat#8487 |
| Experimental Models: Cell Lines |  |  |
| HCT116 | BeNa Culture Collection | BNCC287750 |
| RKO | kmcellbank | KCB2011102YJ |
| SW480 | kmcellbank | KCB200848YJ |
| SW620 | BeNa Culture Collection | BNCC337664 |
| HT-29 | BeNa Culture Collection | BNCC337732 |
| Chemicals |  |  |
| L-Cystine | Sigma-Aldrich | Cat#C6727 |
| L-Cysteine | Sigma-Aldrich | Cat#C7352 |
| L-Glutamine, 200mM Solution | Invitrogen | Cat#25030081 |
| L-Methionine | Sigma-Aldrich | Cat#M5308 |
| L-Glutamic acid monosodium salt hydrate | Sigma-Aldrich | Cat#G5889 |
| L-Aspartic acid sodium salt monohydrate | Sigma-Aldrich | Cat#11195 |
| L-Arginine | Sangon Biotech | Cat#A600205 |
| L-Lysine | Sangon Biotech | Cat#A602759 |
| L-Leucine | Sangon Biotech | Cat#A600922 |
| Earle’s balanced salt solution | Meilun Bio | Cat#MA0031 |
| N-Acetyl-L-cysteine | MedChemExpress | Cat#HY-B0215 |
| L-Glutathione reduced | MedChemExpress | Cat# HY-D0187 |
| Everolimus | MedChemExpress | Cat#HY-10218 |
| Rapamycin | MedChemExpress | Cat#HY-10219 |
| Oxaliplatin | MedChemExpress | Cat#HY-17371 |
| Irinotecan | MedChemExpress | Cat#HY-16562 |
| L-Buthionine-sulfoximine | Dalian Meilun Biotechnology | Cat#MB2690 |
| Sulfasalazine | Sigma-Aldrich | Cat#S0883 |
| Sulforhodamine B sodium salt | Sigma-Aldrich | Cat#S9012 |
| Experimental Models: Organisms |  |  |
| BALB/c nude mice | Hunan Sja Laboratory | N/A |

**Supplementary Table 7. Sequences of RT-PCR primers and siRNA.**

| **Name** | **Gene ID** | **Sequence** |
| --- | --- | --- |
| **RT-PCR primers** | | |
| *SESN2* | 83667 | F: CCTCCTTCGTGTTTGGCTGT |
|  |  | R: CCAGAGTTGTTCAACGGGTC |
| *SLC7A11* | 23657 | F: GTCTGGGTGGAACTCCTCAT |
|  |  | R: CTCCAGCTGACACTCATGCTA |
| *β-actin* | 60 | F: TGACGTGGACATCCGCAAAG |
|  |  | R: CTGGAAGGTGGACAGCGAGG |
| **siRNAs** | | |
| ATF4 | 468 | GAAGGAGTTCGACTTGGAT |
| mTOR 1# | 2475 | GTGCAACCCTTCTTTGACA |
| mTOR 2# |  | GCCGCATTGTCTCTATCAA |
| SLC7A11 1# | 23657 | GGAAGAGATTCAAGTATTA |
| SLC7A11 2# |  | CTTGCAATATGTATATCCA |
